# A rapid and sensitive method for determination of carotenoids in plant tissues by high performance liquid chromatography

**DOI:** 10.1186/s13007-015-0051-0

**Published:** 2015-02-06

**Authors:** Prateek Gupta, Yellamaraju Sreelakshmi, Rameshwar Sharma

**Affiliations:** Repository of Tomato Genomics Resources, Department of Plant Sciences, School of Life Sciences, University of Hyderabad, Hyderabad, 500046 India

**Keywords:** Carotenoids screening, HPLC, C30 column, cis-Isomers, Tomato

## Abstract

**Background:**

The dietary carotenoids serve as precursor for vitamin A and prevent several chronic-degenerative diseases. The carotenoid profiling is necessary to understand their importance on human health. However, the available high-performance liquid chromatography (HPLC) methods to resolve the major carotenoids require longer analysis times and do not adequately resolve the violaxanthin and neoxanthin.

**Results:**

A fast and sensitive HPLC method was developed using a C30 column at 20°C with a gradient consisting of methanol, methyl-*tert*-butyl ether and water. A total of 15 major carotenoids, including 14 all-trans forms and one cis form were resolved within 20 min. The method also distinctly resolved violaxanthin and neoxanthin present in green tissues. Additionally this method also resolved geometrical isomers of the carotenoids.

**Conclusion:**

The HPLC coupled with C30 column efficiently resolved fifteen carotenoids and their isomers in shorter runtime of 20 min. Application of this method to diverse matrices such as tomato fruits and leaves, Arabidopsis leaves and green pepper fruits showed the versatility and robustness of the method. The method would be useful for high throughput analysis of large number of samples.

**Electronic supplementary material:**

The online version of this article (doi:10.1186/s13007-015-0051-0) contains supplementary material, which is available to authorized users.

## Background

Carotenoids have been extensively studied in different matrices to analyze their distribution and levels, as diet rich in carotenoids imparts health benefit properties. They are the most widely distributed pigments in nature [1] and more than 700 different carotenoids have been identified so far [2]. In plants, they act as accessory pigments for photosynthesis and precursor to plant hormone ABA and strigolactones [3]. Though the forms of carotenoids found in foods are not many, however, their composition is very complex and varies both qualitatively and quantitatively. Moreover, biological matrices often contain hundreds to thousands of other plant metabolites that interfere with detection of carotenoids [4].

Carotenoids are made up of polyene hydrocarbon chain consisting of eight isoprene units and are classified into two groups: hydrocarbons (carotenes) and their oxygenated derivatives (xanthophylls). The presence of conjugated double bonds and cyclic groups at ends leads to the formation of variety of stereoisomers. In nature, carotenoids exist as cis/trans isomer, however, they exist primarily in the more stable all-trans isomeric form, but cis isomers do occur. Different carotenoids vary significantly in their absorption maxima as well as in their fine structure. Absorption spectra of carotenoids are unique, mostly showed three proximately distinct peaks [5]. The ratio of absorption peak heights from the trough between peak II and III is used for distinguishing carotenoids and their isomers. The isomers can also often be tentatively identified by the presence of a “cis peak”.

Animals are unable to synthesize carotenoids and acquire them from plants through diet. The carotenoid composition of plants is affected by several factors such as cultivar or variety; part of the plant; stage of maturity; climate or geographic site of production; harvesting and postharvest handling; processing and storage [6]. During food processing, the levels of cis-isomers increase due to the isomerization of the trans-isomers. Consequently, effective quantitation of carotenoids in both foods and biological samples is necessary to understand their importance in body metabolism and health.

A variety of methods have been employed to detect the carotenoids in food samples ranging from thin layer chromatography, to high pressure liquid chromatography (HPLC) and combination of HPLC with mass spectrometry including MALDI-TOF. The most commonly used method for identification and quantification of carotenoids utilizes HPLC combined with UV–vis absorption detection. Though carotenoid separation can be carried out using both normal phase and reverse phase HPLC, however, normal phase HPLC is not suitable for carotenoid separation due to poor separation of non-polar carotenoids. In contrast, reverse phase HPLC enables a significant increase in the interaction between analyte and non-polar stationary phase leading to enhanced resolution of carotenoids [7]. Among the columns, C18 columns with isocratic or gradient mode are preferred for carotenoid separation [8]. However, C18 column do not resolve geometrical isomers and inefficiently resolves positional isomers, particularly lutein and zeaxanthin. To maximize chromatographic resolution and selectivity, Sander et al. [9] developed a non-endcapped RP-HPLC column with triacontyl (C30) ligands to resolve carotenoids and its isomers. The polymeric C30 columns also possess abilities to resolve cis/trans-carotenoids [10]. Nevertheless, the efficiency of C30 column to resolve geometrical isomers of carotenoids is offset by requirement of longer run times needing 60 minutes or more for complete separation of carotenoids resulting in low throughput.

Recently, UHPLC technology has been used to analyze carotenoids in various matrices [11]. UHPLC offers several advantages over HPLC such as higher peak capacities, smaller peak widths, gain in sensitivity and higher chromatographic resolution. The shorter analysis times also considerably save mobile phase solvents. Since C30 stationary phase columns are not commercially available for UHPLC, C18 columns have been used for carotenoid separation despite the limitation that C18 columns poorly resolve carotenoid isomers.

In a recent study, C18 UHPLC columns were compared with a C30 HPLC column. Though usage of C30 HPLC column resulted in better resolution of carotenoids, particularly of geometrical isomers compared to C18 UHPLC columns, this advantage was negated by longer run time needed for C30 HPLC column (100 min versus 23 min for C18 UHPLC column) [12]. In another study, Maurer et al. [13], demonstrated the separation of 11 major carotenoids in 13.50 minutes using C18 column on UHPLC-UV for faster analysis. However, using this method they could detect only few geometrical isomers. Though currently available UHPLC methods allow shorter run times, these methods are poor in resolving cis isomers of different carotenoids.

Supercritical fluid chromatography (SFC) and comprehensive two-dimensional LC (LC × LC) has also been applied for the separation of carotenoids, to enhance the chromatographic separations of complex carotenoid mixtures [2]. Although both techniques have better potential to carry out complex carotenoid separations, nonetheless require more specialized and expensive instruments and longer analysis times nearly double than HPLC. The longer analysis times also necessitate special precautions to avoid carotenoid degradation.

The availability of a rapid HPLC method using a polymeric C30 column would greatly aid separation, identification and quantitation of various carotenoid isomers with better sensitivity and selectivity. In this study, we developed a rapid HPLC method using C30 column to determine the carotenoid profiles along with identification of carotenoid isomers from different plant organs. The method is also capable of separation and quantitation of the major carotenoids present in fruit and green leaves.

## Results and discussion

### HPLC analysis of carotenoids standards

It is well known that the separation of carotenoids is strongly influenced by the properties of stationary phase. Among the stationary phases used, polymeric, non-endcapped stationary phases with C30 ligands give optimal separation of carotenoids and their isomers. In contrast, C18 stationary phases are of insufficient thickness to allow full penetration of carotenoid molecules leading to poor isomer separation due to weak solute-bonded phase interactions. It is by virtue of these properties a C30 column provides better resolution of carotenoids and their geometrical isomers than a C18 column [14]. Using a C30 column in HPLC, Fraser et al. [15] separated carotenes, xanthophylls, ubiquinones, tocopherols, and plastoquinones in a single run, however, their analysis time was 42 minutes. Since a longer run time is a major drawback for high throughput analysis, we improved the analytical method by modifying solvent percentages of mobile phase to reduce the run time.

Nelis and de Leenheer [14] advocated the use of nonaqueous reversed-phase liquid chromatography for the separation of complex carotenoid mixtures, citing optimal sample solubility. This results in minimum risk of sample precipitation on the column, increased sample capacity, excellent chromatographic efficiency, and prolonged column life. Though carotenoids are sparingly soluble in water, most studies employ solvent mixtures containing a small fraction of water. In this study, a small fraction of water was used to resolve the early eluting free xanthophylls, such as those present in leaves and mature green fruits. In initial optimization phase, when we used binary solvents containing (B) methanol/ water (95:5, v/v) and (C) *tert*-methyl butyl ether, lutein coeluted with chlorophyll b marring the chromatographic resolution and analyte identification. In successive trials by introducing new steps in the gradient, the relative separation was considerably increased. Of all the gradients tested, the best separation was achieved with (A) methanol/water (98:2) and (C) MTBE for initial 2 minutes, which clearly resolved all-trans-violaxanthin and all-trans-neoxanthin; lutein and Chl b peaks, followed by next 10 minutes run with solvent (B) methanol/water (95:5, v/v) and (C) *tert*-methyl butyl ether. The separation of chlorophylls from the carotenoids also eliminated the need for saponification of samples for removal of chlorophylls.

The optimization of carotenoid separation needs compatibility between injection solvent and mobile phase. Ideally the injection solvent should be either compatible with the mobile phase or more polar than the reverse phase to provide on-column concentration of samples. In case carotenoids are more soluble in the injection solvent than in the mobile phase, and especially when solution is nearly saturated, the carotenoids will precipitate on injection, leading to peak tailing. Alternatively, they will remain in the injection solvent while passing through the column, resulting in broad bands and doubled peaks [16]. On the other hand, the sample will not dissolve fully in mobile phase if the injection solvent is too weak.

In order to improve resolution, we tried different injection solvents like dichloromethane, tetrahydrofuran, ethyl acetate, acetone, and different ratios of mobile phase solvents (MeOH and MTBE). We found that the mixture of mobile phase solvents (MTBE and MeOH) is most ideal for injections of standards and samples in the column. Our analysis revealed 2:3 ratio of MTBE:MeOH is ideal for green tissues whereas 3:1 ratio of MTBE:MeOH is best for red ripe tomato fruits (Additional file 1: Figure S1). The usage of two different ratios was related to difference in composition of carotenoids in green tissues and tomato fruits respectively. As green tissues are rich in xanthophylls, the high content of methanol in injection solvent improves the resolution of early eluting xanthophylls specifically all-trans-violaxanthin and all-trans-neoxanthin. The red ripe tomato fruits are enriched in lycopene, therefore higher content of MTBE is required to completely dissolve lycopene.

For optimal resolution of carotenoids, it is established that column temperature is an important factor in improving separations and reproducibility of retention times, which is critical for correct identification of peaks in complex mixtures. The lower temperatures (ca. 13°C) maximize selectivity for a set of cis/trans isomers, whereas high temperatures (i.e. 38°C) efficiently resolve different carotenoids [17]. At lower temperature, the carotenoids like lutein, zeaxanthin, β-carotene and lycopene were better resolved, while separation of echinenone and α-carotene improved as the temperature increased [16]. Bohm [18] reported that a column temperature of 23 ± 1°C seems to be the best compromise for the separation of most prominent carotenoids, including their cis isomers. Considering the above, we examined carotenoid separation at 12°C, 18°C, 20°C and 25°C. Amongst them, the maximum selectivity was obtained at 20°C column temperature that was used for all other optimization.

**Figure 1:**
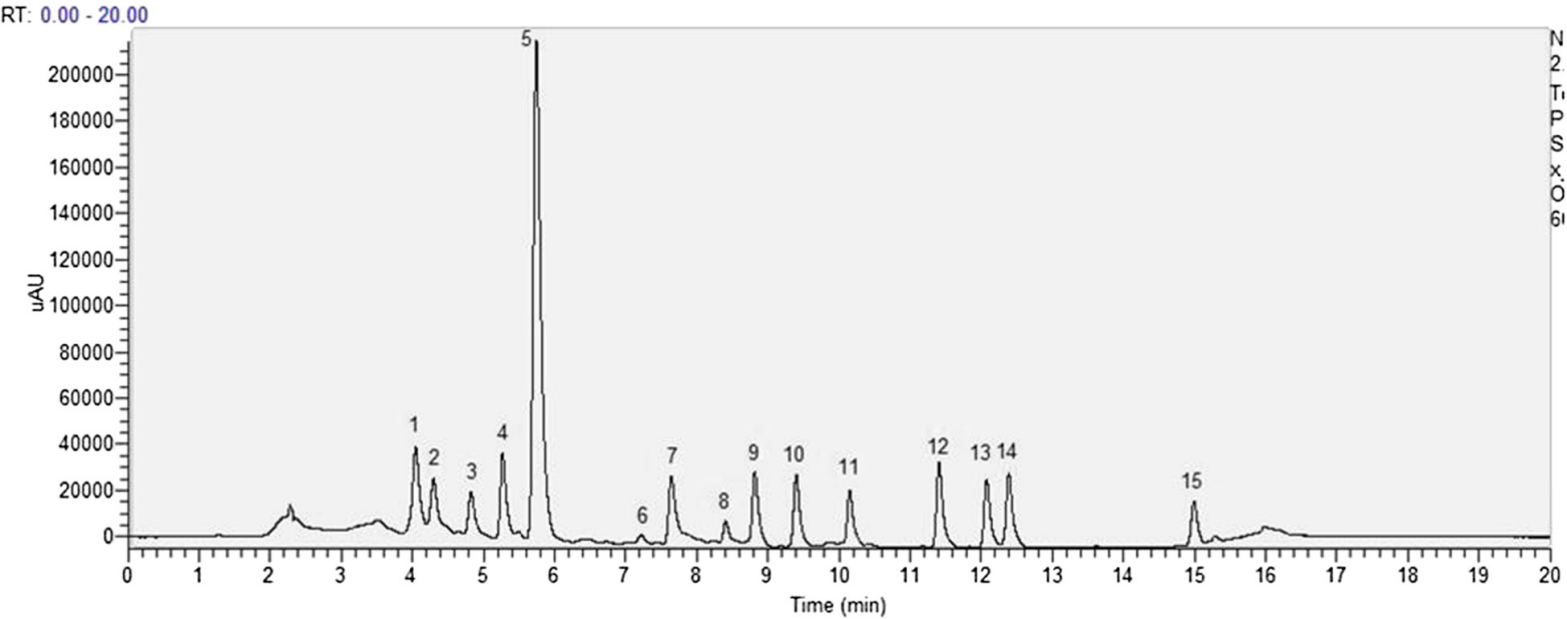
**HPLC profile of carotenoid standards recorded in the range of 250.00-700.00 nm.** The compounds are (1) violaxanthin; (2) neoxanthin; (3) anthraxanthin; (4) lutein; (5) zeaxanthin; (6) phytoene; (7) β-cryptoxanthin; (8) phytofluene; (9) α-carotene; (10) β-carotene; (11) ζ-carotene; (12) δ-carotene; (13) γ-carotene; (14) neurosporene; (15) lycopene.

After careful evaluation of different mobile phases/conditions, a gradient mobile phase consisting of methanol, water and methyl-tert butyl ether, as described in methods section was selected for the analysis of carotenoids and their isomers. Figure 1 shows the chromatogram of carotenoids standards mix. In a run time of 20 minutes, fifteen major carotenoids were separated and identified. The elution profile indicates that good separation efficiency along with shorter separation time was achieved for carotenoid analysis. Table 1 presents the chromatographic and the quantification data for the carotenoids. To assess the solvent strength of the mobile phase the *k* value (retention factor) is used. The *k* values of all peaks ranged between 1.66 and 8.92, indicating that a proper solvent strength of the mobile phase was maintained. Generally, it is accepted that for optimum separation, the *k* value should range from 2 to 10, however, when complicated mixture of compounds are to be separated it can range between 0.5 and 20 [19]. The separation or selectivity factor (*α*) values describes the separation of two species on a column. The selectivity factor is always greater than one. When α is close to unity, *k* is optimized first and then α is increased by changing the mobile phases, column temperature or composition of stationary phase. In our results, for all the peaks, the α were greater than 1.0, implying that a good selectivity of mobile phase to sample components was achieved.

**Table 1:** **Identification and chromatographic data of carotenoid standards**

**Peak no.**	**Compound**	**RT (min)**	**λ (nm) found**	**%III/II found**	**Regression equation**	**R** ^**2**^	**LOD**	**LOQ**	***k***	**α**	**%CV**
**1**	All-trans-violaxanthin	4.05	416.0, 439.0, 469.0	88.8	y = 1.011*x* - 0.270	0.991	0.075	0.250	1.66	1.10	5.45
**2**	All-trans-neoxanthin	4.31	415.0, 437.0, 465.0	78.2	y = 1.042*x −*1.842	0.996	0.075	0.250	1.83	1.17	5.30
**3**	All-trans-antheraxanthin	4.80	(424.0), 446.0, 474.0	60.0	y = 1.057*x −*1.919	0.994	0.075	0.250	2.15	1.13	4.94
**4**	All-trans lutein	5.25	(424.0), 445.0, 474.0	62.5	y = 1.000*x* - 0.040	0.997	0.075	0.250	2.45	1.12	4.43
**5**	All-trans-zeaxanthin	5.73	(428.0), 451.0, 478.0	40.0	y = 1.046*x* - 1.725	0.998	0.075	0.250	2.76	1.37	5.66
**6**	15-cis-phytoene	7.29	(277.0), 286.0, (298.0)	n.c.	y = 1.066*x* - 1.081	0.989	0.120	0.400	3.79	1.06	7.74
**7**	All-trans- β-cryptoxanthin	7.66	(424.0), 452.0, 479.0	35.0	y = 1.028*x* - 1.282	0.991	0.075	0.250	4.03	1.12	6.84
**8**	All-trans-phytofluene	8.43	332.0, 348.0, 367.0	90.9	y = 1.001*x* - 0.118	0.998	0.120	0.400	4.54	1.06	6.60
**9**	All-trans- α-carotene	8.84	424.0, 446.0, 475.0	63.3	y = 1.012*x* - 0.892	0.991	0.075	0.250	4.81	1.07	7.94
**10**	All-trans- β-carotene	9.42	(425.0), 452.0, 479.0	30.0	y = 1.016*x* - 1.176	0.990	0.075	0.250	5.19	1.09	6.36
**11**	All-trans- ζ-carotene	10.18	380.0, 403.0, 426.0	115.4	y = 1.015*x* - 1.092	0.991	0.075	0.250	5.69	1.14	7.69
**12**	All-trans- δ-carotene	11.43	432.0, 457.0, 488.0	71.4	y = 1.014*x* - 1.015	0.989	0.075	0.250	6.51	1.06	5.22
**13**	All-trans- γ-carotene	12.09	439.0, 462.0, 492.0	53.3	y = 1.008*x* - 0.396	0.983	0.075	0.250	6.95	1.02	8.40
**14**	All-trans-neurosporene	12.40	416.0, 440.0, 469.0	92.3	y = 1.010*x* - 1.041	0.991	0.075	0.250	7.15	1.25	4.86
**15**	All-trans-lycopene	15.09	446.0, 472.0, 503.0	73.9	y = 1.027*x* - 0.630	0.993	0.075	0.250	8.92	1.37	10.67

Parentheses indicate a shoulder.

%III/II represents the ratio of peak heights from the trough between peak II and III.

For extraction of carotenoids from plant samples, mixture of different extraction solvents comprising diethyl ether: chloroform (1:2), methanol: chloroform: dichloromethane (1:2:1), methanol: chloroform: acetone (1:2:1) and dichloromethane: chloroform (1:2) were evaluated. Of these, a better resolution and recovery was obtained with dichloromethane: chloroform (1:2), as it does not contain methanol, therefore it also eliminates the metabolites interfering with the detection of carotenoids.

### Photoisomerization of standards

**Figure 2:**
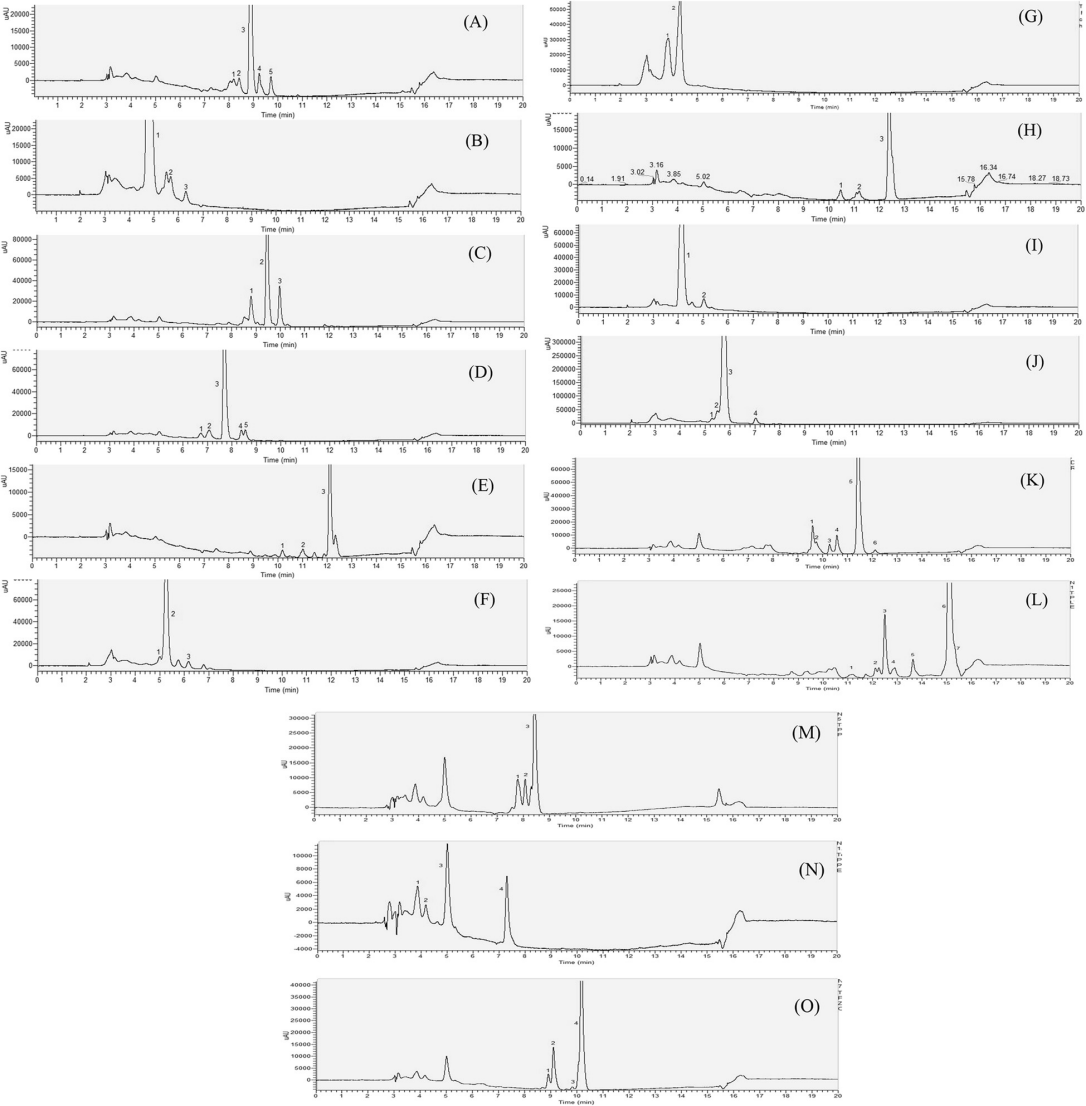
**HPLC chromatogram of different carotenoids and their isomers after photoisomerization. (A)** α-carotene, **(B)** antheraxanthin, **(C)** β-carotene, **(D)** β-cryptoxanthin, **(E)** γ-carotene, **(F)** lutein, **(G)** neoxanthin, **(H)** neurosporene, **(I)** violaxanthin, **(J)** zeaxanthin, **(K)** δ-carotene, **(L)** lycopene, **(M)** phytofluene, **(N)** phytoene and **(O)** ζ-carotene. The Arabic numeral in bold preceding carotenoid isomer refers to its peak on chromatogram. **(A)**: 1; 13-cis-α-carotene; 2; 13′-cis-α-carotene; 3; all-trans-α-carotene; 4; 9-cis-α-carotene; 5; 9′-cis-α-carotene. **(B)**: 1; all-trans-antheraxanthin; 2; 9-cis-antheraxanthin; 3; 9′-cis-antheraxanthin. **(C)**: 1; 13-cis-β-carotene; 2; all-trans-β-carotene; 3; 9-cis-β-carotene. **(D)**: 1; 13-cis-β-cryptoxanthin; 2; 13′-cis-β-cryptoxanthin; 3; all-trans-β-cryptoxanthin; 4; 9-cis-β-cryptoxanthin; 5; 9′-cis-β-cryptoxanthin. **(E)**: 1; cis-γ-carotene 2; cis-γ-carotene 3; all-trans-γ-carotene. Lutein **(F)**: 1; 13- or 13′-cis-lutein; 2; all-trans-lutein; 3; 9- or 9′-cis-lutein. Neoxanthin **(G)**: 1; 9-cis-neoxanthin; 2; all-trans-neoxanthin. Neurosporene **(H)**: 1; 15-cis-neurosporene; 2; 13-cis-neurosporene; 3; all-trans-neurosporene. Violaxanthin **(I)**: 1; all-trans-violaxanthin; 2; 9-cis-violaxanthin. Zeaxanthin **(J)**: 1; 15-cis-zeaxanthin; 2; 13-cis-zeaxanthin; 3; all-trans-zeaxanthin; 4; 9-cis-zeaxanthin. δ-carotene **(K)**: 1; δ-carotene isomer 1, 2; δ-carotene isomer 2, 3; δ-carotene isomer 3, 4; δ-carotene isomer 4, 5; all-trans-δ-carotene, 6; δ-carotene isomer 5. Lycopene **(L)**: 1; di-cis-lycopene 1, 2; di-cis-lycopene 2, 3; 15-cis-lycopene, 4; 13-cis-lycopene, 5; 9-cis-lycopene, 6; all-trans-lycopene, 7; 5-cis-lycopene. Phytofluene **(M)**: 1; 15,9′-cis-phytofluene, 2; phytofluene isomer, 3; all-trans-phytofluene. Phytoene **(N)**: 1; phytoene isomer 1, 2; phytoene isomer 2, 3; phytoene isomer 3, 4; all-trans-phytoene. ζ-carotene **(O)**: 1; 9.15,9′ cis- ζ-carotene, 2; ζ-carotene isomer 1, 3; ζ-carotene 2, 4; all-trans-ζ-carotene. The HPLC profiles were recorded in the range of 250.00-700.00 nm.

For identification of cis-carotenoids, the fifteen carotenoid standards were illuminated to accelerate cis isomers formation by using a procedure described in the methods section. The retention time and absorption spectral characteristics of carotenoid isomers were used in identifying the unknown peaks in the samples. Figure 2 shows the HPLC chromatograms of photoisomerized carotenoid standards, including α-carotene (Figure 2A), antheraxanthin (Figure 2B), β-carotene (Figure 2C), β-cryptoxanthin (Figure 2D), γ-carotene (Figure 2E), lutein (Figure 2F), neoxanthin (Figure 2G), neurosporene (Figure 2H), violaxanthin (Figure 2I), zeaxanthin (Figure 2J), δ-carotene (Figure 2K), lycopene (Figure 2L), phytofluene (Figure 2M), phytoene (Figure 2N) and ζ-carotene (Figure 2O). Table 2 represents the chromatographic and quantification data for the cis isomers of carotenoids. The identification of the cis isomers was based on the cis peak, wavelength spectrum and Q ratio’s (ratio of the height of the cis-peak to the main absorption peak) with those in the literature. The retention time and absorption spectral characteristics of carotenoid isomers were used for identifying the unknown peaks in the samples. The on-line PDA spectra of all-trans and isomerized standards are presented in Additional file 2: Figure S2.

**Table 2:** **Tentative identification of photoisomerised isomers after illumination of all-trans standards**

**S. no.**	**Compound**	**RT (min)**	**λ (nm) found**	**λ (nm) reported**	***Q*** **ratio found**	***Q*** **ratio reported**	**%III/II**	**Ref.**
**1**	9-cis-neoxanthin	3.78	328.0,(418.0),439.0, 468.0	327.0,416.0, 439.0, 468.0	0.13	0.16	55.55	[20]
**2**	Phytoene isomer 1	3.89	277.0					
**3**	Phytoene isomer 2	4.18	265.0, 272.0				19.08	
**4**	13-cis or 13′-cis-Lutein	5.00	330.0,(420.0),441.0, 466.0	332.0,416.0, 440.0, 468.0	0.39	0.39	23.07	[21]
**5**	Phytoene isomer 3	5.02	270.0					
**6**	9-cis-violaxanthin	5.03	327.0, 412.0, 435.0, 464.0	328.0,412.0, 436.0, 464.0	0.12	0.11	87.5	[22]
**7**	15-cis zeaxanthin	5.25	335.0, (422.0), 447.0, 470.0	338.0,422.0, 446.0, 470.0	0.48	0.45		[23]
**8**	13-cis zeaxanthin	5.49	337.0, (422.0), 444.0, 469.0	338.0,424.0, 446.0, 472.0	0.43	0.37	8.69	[23]
**9**	9-cis-antheraxanthin	5.68	330.0, (419.0), 442.0, 469.0	332.0,440.0, 468.0	0.13	0.09	5.33	[24]
**10**	Phytoene isomer	5.88	(276.0), 286.0, (298.0)					
**11**	9-cis or 9′-cis-Lutein	6.18	333.0, (419.0), 440.0, 468.0	332.0,416.0, 440.0, 470.0	0.08	0.13	64.70	[21]
**12**	9′-cis-antheraxanthin	6.29	330.0, (419.0), 441.0, 468.0	332.0,440.0, 468.0	0.07	0.08	53.57	[24]
**13**	13-cis-β-cryptoxanthin	6.73	336.0, (421.0), 449.0, 473.0		0.25			
**14**	9-cis-zeaxanthin	7.02	341.0, (422.0), 446.0, 473.0	338.0,422.0, 446.0, 474.0	0.11	0.12	36.36	[23]
**15**	13′-cis β-cryptoxanthin	7.08	336.0, (418.0), 444.0, 469.0	336.0,415.0, 443.0, 470.0	0.43	0.47	10.43	[25]
**16**	15,9′-cis-phytofluene	7.81	332.0, 348.0, 367.0	330.0, 347.0, 366.0			71.42	[26]
**17**	Phytofluene isomer 1	8.08	332.0, 348.0, 367.0				82.92	
**18**	13-cis-α-carotene	8.19	331.0, 418.0, 440.0, 466.0	330.0,417.0, 438.0, 466.0	0.43	0.43	33.33	[26,27]
**19**	9-cis-β-cryptoxanthin	8.38	336.0, (420.0), 446.0, 473.0	339.0,418.0, 445.0, 472.0	0.10	0.16	33.33	[25]
**20**	13′-cis α-Carotene	8.41	329.0, 416.0, 439.0, 466.0	332.0,416.0, 438.0, 465.0	0.35	0.41	33.33	[27]
**21**	9′-cis β-cryptoxanthin	8.55	338.0, (421.0), 447.0, 473.0	339.0,420.0, 445.0, 472.0	0.08	0.13	36.36	[25]
**22**	15-cis β-carotene	8.80	338.0, (420.0), 444.0, 468.0	337.0,420.0, 444.0, 470.0	0.42	0.41		[10]
**23**	9,15,9′ cis-ζ-carotene	8.90	295.0, 378.0, 397.0, 423.0	296.0, 377.0, 399.0, 424.0	0.34	0.24	76.92	[28]
**24**	ζ-carotene isomer 1	9.10	296.0, 375.0, 395.0, 420.0	276.0, 375.0, 395.0, 420.0	0.25	0.23	86.30	[28]
**25**	9-cis α-carotene	9.24	330.0, 419.0, 441.0, 469.0	330.0,418.0, 441.0, 467.0	0.08	0.1	90.90	[26,27]
**26**	δ-carotene isomer 1	9.60	345.0, 425.0, 450.0, 480.0		0.48		53.12	
**27**	9′-cis α-Carotene	9.71	330.0, 421.0, 442.0, 469.0	330.0,421.0, 441.0, 469.0	0.12	0.08	50.00	[27]
**28**	δ-carotene isomer 2	9.75	345.0, 428.0, 453.0, 483.0	349.0, 430.0, 453.0, 482.0	0.54	0.42	42.85	[20]
**29**	ζ-carotene isomer 2	9.84	379.0, 401.0, 426.0	380.0, 401.0, 426.0			112.90	[28]
**30**	9-cis-β-carotene	9.97	346.0, (420.0), 447.0, 473.0	335.0, 421.0, 447.0, 472.0	0.10	0.09	31.80	[20,29]
**31**	Cis γ-carotene 1	10.18	348.0, 432.0, 454.0, 481.0		0.44		15.38	
**32**	δ-carotene isomer 3	10.27	426.0, 451.0, 481.0				71.42	
**33**	15-cis-neurosporene	10.46	330.0, 412.0, 435.0, 463.0	464.0	0.47	0.48	59.09	[30]
**34**	δ-carotene isomer 4	10.58	346.0, 425.0, 450.0, 480.0		0.39		64.51	
**35**	Cis γ-carotene 2	11.00	348.0, 433.0, 455.0, 486.0		0.20		40.00	
**36**	13-cis-neurosporene	11.20	330.0, 412.0, 434.0, 462.0	461.0	0.18	0.11	79.16	[30]
**37**	di-cis-lycopene 1	11.20	434.0, 457.0, 488.0	350.0, 458.0			57.0	[19]
**38**	di-cis-lycopene 2	12.10	460.0, 488.0	350.0, 464.0, 488.0	0.25		12.5	[19]
**39**	δ-carotene-isomer 5	12.14	428.0, 453.0, 484.0				73.07	
**40**	15-cis-lycopene	12.51	361.0, 440.0, 467.0, 495.0	360.0, 437.0, 466.0, 494.0	0.57	0.75	18.75	[19]
**41**	13-cis-lycopene	12.83	361.0, 442.0, 465.0, 495.0	360.0, 437.0, 463.0, 494.0	0.58	0.55	35.71	[31]
**42**	9-cis-lycopene	13.64	440.0, 467.0, 497.0	360.0, 438.0, 464.0, 494.0	0.27	0.13	66.66	[31]
**43**	di-cis-lycopene 3	14.19	446.0, 472.0, 503.0	446.0, 472.0, 503.0	0.15	0.08	70.58	[31]
**44**	5-cis-lycopene	15.08	446.0, 472.0, 503.0	362.0, 442.0, 470.0, 502.0		0.06	74.28	[31]

Parentheses indicate a shoulder.

Q-ratio is the height ratio of the cis-peak to the main absorption peak.

%III/II represents the ratio of peak heights from the trough between peak II and III.

### Method validation

As mentioned in the above section, carotenoid standard curves were prepared for subsequent quantitation by HPLC–PDA. The amounts of carotenoids were calculated from the regression equations presented in Table 1. The limit of detection (LOD) and limit of quantification (LOQ) were calculated for each standard (Table 1). The limit of detection (LOD) was defined as the amount that resulted in a peak with a height three times that of the baseline noise respectively and the limit of quantification (LOQ) was determined as lowest injected amount which could be quantifiable reproducibly (RSD ≤ 5%). The precision was evaluated by the relative CV (%CV) which ranges from 4.43-10.67 (Table 1). The intra-day relative standard deviations (R.S.D.) were 0.008–0.02% for retention times of individual carotenoid and 0.54–2.13% for standard concentrations, whereas the inter-day R.S.D. were 0.04–0.08% for retention times and 1.13–3.97% for standard concentrations, demonstrating that a high reproducibility was achieved by using this method.

The accuracy of the extraction method was assessed by determining recovery of all-trans violaxanthin, neoxanthin, antheraxanthin, lutein, zeaxanthin, β-cryptoxanthin, α-carotene, β-carotene, ζ-carotene, δ-carotene, γ-carotene, neurosporene, lycopene, 15-cis-phytoene and all-trans phytofluene, with a mean value of 82.1, 93.3, 81.0, 86.5, 92.4, 83.2, 98.0, 80.0, 92.1, 82.2, 88.7, 98.0, 93.6, 94.4 and 94.6% being attained, respectively.

### Application of the method to various plant tissues

**Figure 3:**
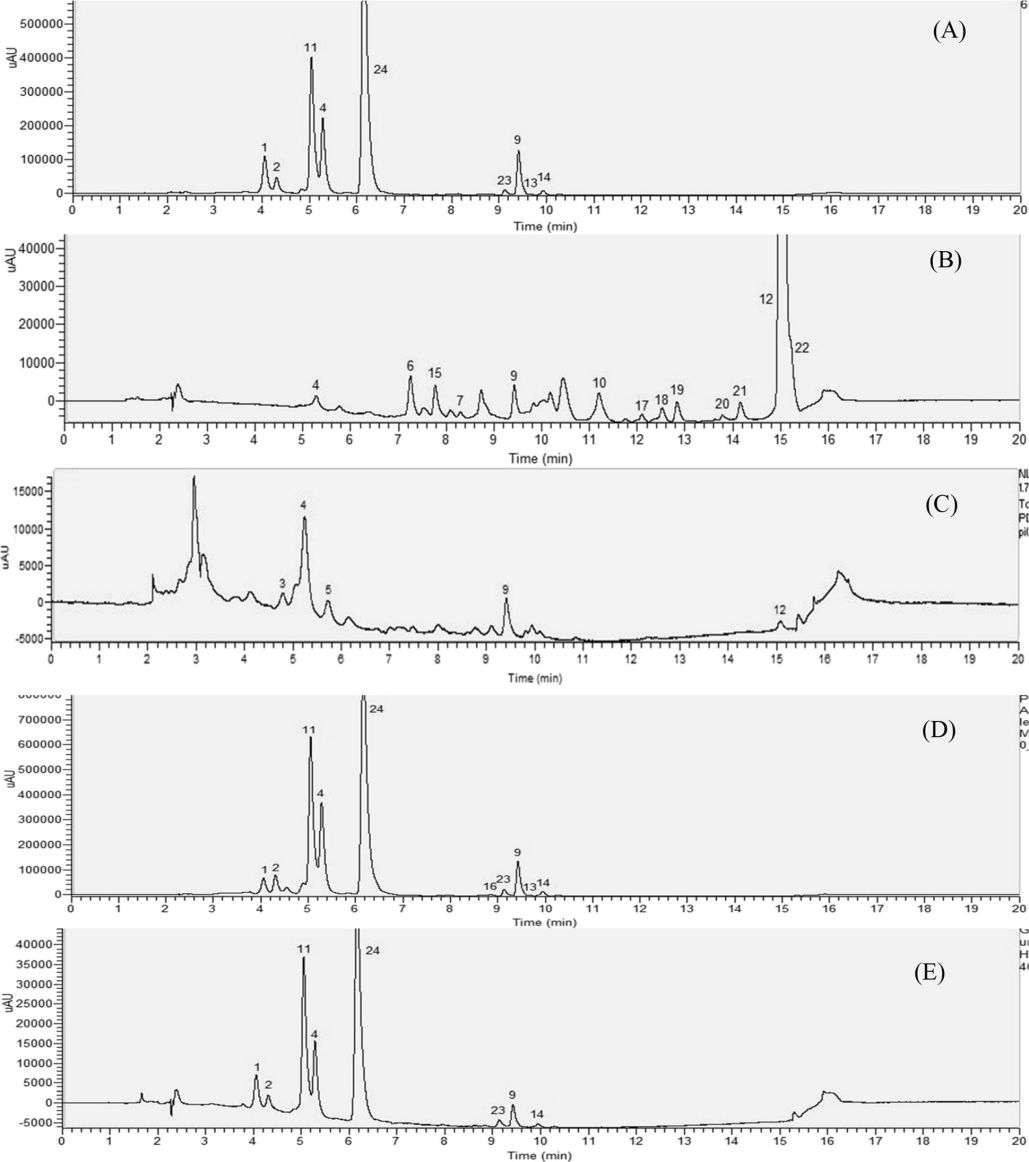
**HPLC profiles of the major carotenoids in tomato leaf (A), tomato red ripe fruits (B), yellow fruited ripe tomato (PI114490) (C), Arabidopsis leaf (D) and green capsicum (E).** Peak identification: (1) trans-violaxanthin, (2) trans-neoxanthin, (3) trans-antheraxanthin, (4) trans-lutein, (5) trans-zeaxanthin, (6) 15-cis-phytoene, (7) 15,9′-cis-phytofluene, (9) all-trans-β-carotene, (10) di-cis-lycopene 1 (11) chlorophyll b, (12) all-trans-lycopene, (13) 9′-cis-α-carotene, (14) 9-cis-β-carotene, (15) all-trans-phytofluene, (16) 15-cis-β-carotene, (17) di-cis-lycopene 2, (18) 15-cis-lycopene, (19) 13-cis-lycopene, (20) 9-cis-lycopene, (21) di-cis-lycopene 3, (22) 5-cis-lycopene, (23) chlorophyll a and (24) pheophytin b. The HPLC profiles were recorded in the absorbance range of 250.00-700.00 nm.

To check the versatility of the method, carotenoid content was estimated from leaf and red ripe fruit tissue of field grown Indian tomato cultivar Arka Vikas. Carotenoids were also estimated from Arabidopsis leaf, and green capsicum fruits purchased from the local market. The extracts were analyzed using the C30 column on HPLC. In tomato leaves, three xanthophylls, lutein (26.82 μg/g FW), violaxanthin (14.27 μg/g FW) and neoxanthin (17.58 μg/g FW) and β-carotene (27.02 μg/g FW) as the principal carotene were present. In tomato leaves, other carotenoids were 9′-cis-α-carotene (1.08 μg/g FW) and 9-cis-β-carotene (4.36 μg/g FW). Tomato fruit tissue contains lutein (3.05 μg/g FW) as xanthophylls and all-trans-lycopene (66.2 μg/g FW) is the major carotenoid present along with β-carotene (5.10 μg/g FW), carotenoid pathway precursors phytoene (8.46 μg/g FW) and phytofluene (1.23 μg/g FW). Using our chromatographic conditions, several cis isomers of lycopene were separated which cannot be resolved by C18 columns. The isomers of lycopene are of interest with respect to their dietary absorption and health beneficial effects [32]. This was also illustrated by the analysis of extracts of the yellow fruited mutant of tomato (PI114490), which contains negligible amounts of carotenoids [33]. The chromatogram of Arabidopsis leaf and green capsicum was similar to the tomato leaf with respect to carotenoid compounds, however the carotenoid content differed. In Arabidopsis leaf 15-cis-β-carotene (3.68 μg/g FW) was also detected. The chromatogram and carotenoid content are presented in Figure 3 and Table 3. We also checked the carotenoid content of tomato tangerine mutant fruits and the major carotenoid compounds 7,9,7′,9′-tetra-cis-lycopene, 9,9′-di-cis-zeta-carotene and 7,9,9′-cis-neurosporene (data not shown) were separated.

**Table 3:** **Concentration (μg/g FW) of all-trans and cis- carotenoids in tomato cultivar Arka Vikas (AV) leaf and red ripe (RR) fruit, Arabidopsis leaf, tomato mutant line-PI 114490 ripe fruit and green capsicum**

**S. no.**	**Compound**	**AV leaf**	**AV RR fruit**	**PI 114490**	**Arabidopsis leaf**	**Green capsicum**
**1**	All-trans-violaxanthin	14.27 ± 0.78	-	-	13.48 ± 1.08	2.75 ± 0.24
**2**	All-trans-neoxanthin	17.58 ± 1.66	-	-	32.77 ± 5.78	2.82 ± 0.22
**3**	All-trans-antheraxanthin	-	-	0.91 ± 0.08	-	-
**4**	All-trans-lutein	26.82 ± 3.87	3.05 ± 0.6	1.25 ± 0.17	67.40 ± 7.23	3.90 ± 0.54
**5**	All-trans-zeaxanthin	-	-	0.28 ± 0.02	-	-
**6**	15-cis-phytoene	-	8.46 ± 1.15	-	-	-
**7**	15,9′-cis-phytofluene	-	1.23 ± 0.31	-	-	-
**8**	All-trans-β-carotene	27.02 ± 3.48	5.10 ± 1.08	0.64 ± 0.2	62.05 ± 9.80	3.60 ± 0.43
**9**	All-trans-γ-carotene	-	1.47 ± 0.30	-	-	-
**10**	All-trans-lycopene	-	66.20 ± 4.00	0.37 ± 0.02	-	-
**11**	9′-cis-α-carotene	1.08 ± 0.28	-	-	1.97 ± 0.63	-
**12**	9-cis-β-carotene	4.36 ± 0.80	-	-	3.45 ± 0.96	-
**13**	Phytofluene isomer	-	2.42 ± 0.54	-	-	-
**14**	15-cis-β-carotene	-	-	-	3.68 ± 0.86	-
**15**	di-cis-lycopene 1	-	2.39 ± 0.37	-	-	-
**16**	di-cis-lycopene 2	-	1.47 ± 0.54	-	-	-
**17**	15-cis-lycopene	-	2.22 ± 0.45	-	-	-
**18**	13-cis-lycopene	-	1.54 ± 0.60	-	-	-
**19**	9-cis-lycopene	-	1.67 ± 0.37	-	-	-
**20**	di-cis-lycopene 3	-	1.76 ± 0.42	-	-	-
**21**	5-cis-lycopene	-	8.75 ± 1.08	-	-	-

The literature is unanimous in reporting the efficiency of a polymeric C30 column over C18 column in resolving cis-isomers of carotenoids in fruits/vegetables and biological fluids/tissues [10,19,21,34]. However, there are still many studies using a C18 column for determination of major carotenoids like lutein, zeaxanthin, β-cryptoxanthin, β-carotene, γ-carotene and lycopene [35]. Commonly encountered drawbacks in these studies include poor resolution of lutein and zeaxanthin, which are often quantified together, and simultaneous elution of both all-trans- and cis-isomers of lycopene. In the current chromatographic conditions, lutein and zeaxanthin were resolved. Additionally, all trans-lycopene and its 7 cis-isomers were also separated and quantified. Most importantly all xanthophylls present in green photosynthetic tissues including violaxanthin and neoxanthin were distinctly resolved, indicating wide applicability of the developed method. In essence, the present method using C30 column offers a much improved resolution of carotenoids from different plant samples in shorter run times.

The earlier studies using C30 columns reported prolonged run times for adequate resolution of the carotenoids. The main advantage of our method is the improved resolution of carotenoids with significant reduction in chromatographic run time. Rajendran et al. [10] using polymeric C30 column coupled with HPLC separated all-trans- plus cis-carotenoids within 51 minutes. However their separation did not include precursors like phytoene and phytofluene. In a recent study, Hsu et al. [36] identified 30 carotenoids separated within 45 min in human serum. Using plant tissues, Fraser et al. [15] reported the separation of 25 carotenoids including their isomers in 42 min using C30 column. Fantini et al. [37] identified 45 carotenoids including isomers in 72 minutes in tomato fruits.

Considering that reverse phase HPLC takes longer analysis times, a recent study [12], compared UHPLC verses HPLC stationary phases for carotenoid separation. They concluded that while UHPLC analysis is more suited for rapid screening of carotenoids, for analysis of complex mixture, HPLC coupled with C30 columns gives better resolution. The advantage of C30 column was offset by the fact that analysis on this column took about four times longer than UHPLC (100 min versus 23 min, respectively). In another study, Rivera et al. [38] analyzed a mixture of 16 carotenoids by UHPLC–MS within 15 min but the method was not validated using biological samples. In recent study, Maurer et al. [13], separated 11 major carotenoids in 13.5 min, but it did not include δ-carotene and γ-carotene.

## Conclusions

HPLC coupled with a C30 column and a gradient used in this study resolved carotenoids rapidly and efficiently at a scale comparable to that reported for UHPLC with C18 column. In this method 15 carotenoids including their precursors were separated in shorter run time of 20 min and this method was validated using tomato and other plant samples. This method can be applied to determine the levels of cis/trans-carotenoids and their precursors phytoene and phytofluene in complex biological sample matrices. The method also efficiently resolved the carotenoids and xanthophylls from green tissues and red ripe fruits therefore is suitable for carotenoid analysis from wide range of plant samples. In future this method can be adapted to UHPLC on availability of C30 UPLC column with enhanced resolutions.

## Materials and methods

### Standards and solvents

Violaxanthin, neoxanthin, antheraxanthin, lutein, zeaxanthin, phytoene, β-cryptoxanthin, phytofluene, α-carotene, β-carotene, ζ-carotene, δ-carotene, γ-carotene, neurosporene and lycopene were purchased from CaroteNature (Lupsingen, Switzerland). Methanol was purchased from Fisher Scientific (Waltham, MA, USA). Tert-methyl butyl ether (MTBE), chloroform and dichloromethane were purchased from Avantor Performance Materials (Panoli, Gujrat, India), hexane from Sigma chemical Co. (St. Louis, MO, USA) and ethanol from Hayman Ltd. (Essex, USA). All reagents were HPLC grade or higher. A Millipore Milli-Q water purification system was used to obtain high purity of water.

### Standard preparation

Stock solutions of carotenes and xanthophylls were prepared in hexane and ethanol respectively of 0.1 mg/mL. The exact concentration of each stock solution was determined by spectrophotometry using the absorption coefficients A (1%, 1 cm) of the respective carotenoid (Additional file 3: Table S1). After determination of concentration, the standards were evaporated under nitrogen, and solubilized in methanol/MTBE (60/40, v/v) to obtain a final concentration of 5 μg/mL, that was used for HPLC analysis. Individual working solution of each standard was injected in the HPLC system.

### Extraction procedure

Freeze-dried plant sample (~150 mg) was homogenized using a mortar and pestle or an IKA A11 basic grinder (IKA, Staufen, Germany) and to the homogenate 1.5 mL of chloroform:dichloromethane (2:1, v/v) was added. The resultant suspension was mixed for 20 min using a thermomixer at 1000 rpm at 4°C. Thereafter for phase separation, 0.5 mL of 1 M sodium chloride solution was added and contents were mixed by inversion. After centrifugation at 5000 *g* for 10 min the organic phase was collected. The aqueous phase was re-extracted with 0.75 mL of chloroform:dichloromethane (2:1, v/v), centrifuged and again organic phase was collected. Both organic phases were pooled, dried by centrifugal evaporation, re-dissolved in 1 mL of methanol/MTBE (25/75, v/v) for red ripe fruit tissues and re-dissolved in 1 mL and 200 μL of methanol/MTBE (60/40, v/v) for leaf and mature green fruit respectively prior to analysis. A final volume of 20 μL was used for injection into HPLC.

### Isomerization of carotenoid standards

For generation of *cis*-isomers of carotenoids, 1 mL solution (1 μg/mL) each of all-trans forms of violaxanthin, neoxanthin, antheraxanthin, lutein, zeaxanthin, β-cryptoxanthin, α-carotene, β-carotene, γ-carotene and neurosporene was subjected to photoisomerization as described by Rajendran et al. [10]. The tubes containing standards were illuminated with three 30 W fluorescent light tubes (Anchor B22-6500 K, Eurolite International, Kowloon, Hong Kong) for 24 h at 25°C at a distance of 30 cm and light intensity of 2500–3500 lx. Stereomutation of δ-carotene, ζ-carotene, lycopene, phytoene and phytofluene was carried out by heating at 80°C for 60 minutes. Thereafter, the above standards were evaporated to dryness, dissolved in 100 μL MTBE/MeOH (75/25, v/v) and a 20 μL was injected for determination of retention time, absorption spectra and Q-ratio’s.

### HPLC-PDA analysis of carotenoids

Carotenoids were analyzed by reversed phase HPLC, using Thermo ACCELA U-HPLC (Thermo Fisher Scientific, Bremen, Germany) consisting of quaternary pump, an online degasser, a column oven controller and a photodiode array detector (PDA). Carotenoids were separated on a reverse-phase C30, 3 μm column (250 × 4.6 mm) coupled to a 20 × 4.6 mm C30 guard column (YMC Co., Kyoto, Japan) using mobile phases consisting of (A) methanol/ water (98:2, v/v), (B) methanol/ water (95:5, v/v) and (C) tert-methyl butyl ether. The gradient elution used with this column was 80% A, 20% C at 0 min, followed by linear gradient to 60% A, 40% C to 2.00 min at a flow rate of 1.4 mL/min, at 2.01 minute flow rate was changed to 1.00 mL/min with gradient changing to 60% B, 40% C followed by a linear gradient to 0% B, 100% C by 12 min and return to initial conditions by 13.00 min. A re-equilibration (7.00 min) was carried out at initial concentrations of 80% A, 20% C. The column temperature was maintained at 20°C. The eluting peaks were monitored at a range of 250 to 700 nm using PDA. Quantification was performed using Xcalibur software (version 2.2) comparing peak area with standard reference curves.

### Identification and quantification of carotenoids in samples

Peaks were identified by comparing the retention times and UV–Vis spectral data with those of the corresponding standards. In addition, the *cis*-isomers of carotenoids were tentatively identified based on the absorption at near 330 or 360 nm (cis peak), wavelength spectrum and Q ratio’s with reference to photoisomerized carotenoid standards and reported values in the literature.

Concentration of each analyte was calculated from the calibration curve of the corresponding standard. All standard solutions were prepared as described above in standard preparation section. Five-point external standard curves (ranging from 10, 25, 50, 75 and 100 ng) were constructed for the standard mix. Carotenoid concentrations were then calculated using a linear regression *y* = *ax* + *b*, where *y* = concentration and *x* = area of the five-point standard curve. The regression equation and correlation coefficient (*R*^2^) were obtained using Microsoft® Excel 2013.

The cis-isomers of carotenoids were quantified using the standard curves of all-trans carotenoids because of similarity in extinction coefficient [21].

### Validation Procedure

The developed method was validated in terms of separation, linearity, recovery and reproducibility. The retention factor (k) calculated by using the formula k = (t_R_ − t_0_)/t_0_, where t_R_ and t_0_ denote retention time of sample components and sample solvent, respectively. Based on the retention factor of two neighboring peaks (k1 and k2), separation factor (α) was determined by using the formula, α = k2/k1 [39].

For recovery and reproducibility studies, plant sample was spiked with 0.5 μg/mL and 1.25 μg/mL concentration of each standard respectively. The spiked sample was then extracted adopting the method described in section 2.4. After performing HPLC analysis, the recovery of each carotenoid was calculated by R(%) = [(Cs − Cp)/Ca] × 100, where R(%) is percent recovery, Cs is total carotenoid content in the spiked sample, Cp is endogenous carotenoid content in the sample, and Ca is the amount of carotenoid standard added to the sample. The cis isomers of carotenoids were quantified using the recovery of their corresponding all-trans forms. For every sample analyses were performed in triplicates and the mean value was calculated. The reproducibility of this method was ascertained by taking mean of the two spiked concentrations in three replicates on the same day and on three different days. The %CV (percent coefficient of variation) for each carotenoid was calculated by $\%\mathrm{C}\mathrm{V} = \left(\mathrm{S}\mathrm{D}/\overline{\mathrm{X}}\right)*100$ where SD is the standard deviation and $\overline{\mathrm{X}}$ is the mean. The limit of detection (LOD) and the limit of quantification (LOQ) were calculated as described by International Conference on Harmonization [39].

## Additional files

Additional file 1: Figure S1. HPLC profile of carotenoid standards recorded in the range of 250.00-700.00 nm in different injection solvents. **(A)** Standard mix dissolved in MeOH:MTBE (25:75) and **(B)** Standard mix dissolved in MeOH:MTBE (60:40) The compounds are (1) violaxanthin; (2) neoxanthin; (3) anthraxanthin; (4) lutein; (5) zeaxanthin; (6) phytoene; (7) β-cryptoxanthin; (8) phytofluene; (9) α-carotene; (10) β-carotene; (11) ζ-carotene; (12) δ-carotene; (13) γ-carotene; (14) neurosporene; (15) lycopene. Additional file 2: Figure S2. On-line PDA spectra of all-trans and photoisomerised standards. (1) 9′-cis β-cryptoxanthin , (2) 9′-cis α-carotene, (3) 9′-cis antheraxanthin, (4) 9-cis β-cryptoxanthin, (5) 9-cis lutein, (6) 9-cis zeaxanthin, (7) 9-cis α-carotene, (8) 9-cis antheraxanthin, (9) 9-cis β-carotene, (10) 13-cis α-carotene, (11) 13′-cis α-carotene, (12) 13-cis β-cryptoxanthin, (13) 13′-cis β-cryptoxanthin, (14) 13-cis neurosporene, (15) 13 or 13′-cis lutein, (16) 13-cis zeaxanthin, (17) 15-cis neurosporene, (18) 15-cis β-carotene, (19) 15-cis zeaxanthin, (20) all-trans α-carotene, (21) all-trans antheraxanthin, (22) all-trans β-carotene, (23) all-trans β-cryptoxanthin, (24) all-trans δ-carotene, (25) all-trans γ-carotene, (26) all-trans lutein, (27) all-trans lycopene, (28) all-trans neoxanthin, (29) all-trans neurosporene, (30) all-trans violaxanthin, (31) all-trans zeaxanthin, (32) all-trans ζ-carotene, (33) cis- γ-carotene 2, (34) cis- γ-carotene 1, (35) cis-neoxanthin, (36) cis-violaxanthin, (37) phytoene isomer, (38) phytoene, (39) phytofluene isomer (40) phytofluene (41) di-cis lycopene, (42) di-cis lycopene, (43) 15-cis lycopene, (44) 13-cis lycopene, (45) 9-cis lycopene, (46) di-cis lycopene and (47) 5-cis lycopene. Additional file 3: Table S1. Absorption Coefficients of carotenoid standards.

## Abbreviations

AV: Arka Vikas cultivar of tomato
HPLC: High-performance liquid chromatography
LOD: Limit of detection
LOQ: The limit of quantification
MTBE: *tert*-methyl butyl ether
PDA: Photodiode array
RP-HPLC: Reverse phase-HPLC
RSD: Relative standard deviations
UHPLC: Ultra-HPLC
