# Supplementary material for: A rapid and sensitive method for determination of carotenoids in plant tissues by high performance liquid chromatography
Source: Plant Methods. 2015 Feb 6;11:5. doi: 10.1186/s13007-015-0051-0 (PMC4329677; doi:10.1186/s13007-015-0051-0)
Supplement: Additional file 1: Figure S1. — HPLC profile of carotenoid standards recorded in the range of 250.00-700.00 nm in different injection solvents. (A) Standard mix dissolved in MeOH:MTBE (25:75) and (B) Standard mix dissolved in MeOH:MTBE (60:40) The compounds are (1) violaxanthin; (2) neoxanthin; (3) anthraxanthin; (4) lutein; (5) zeaxanthin; (6) phytoene; (7) β-cryptoxanthin; (8) phytofluene; (9) α-carotene; (10) β-carotene; (11) ζ-carotene; (12) δ-carotene; (13) γ-carotene; (14) neurosporene; (15) lycopene. [file 13007_2015_51_MOESM1_ESM.docx]

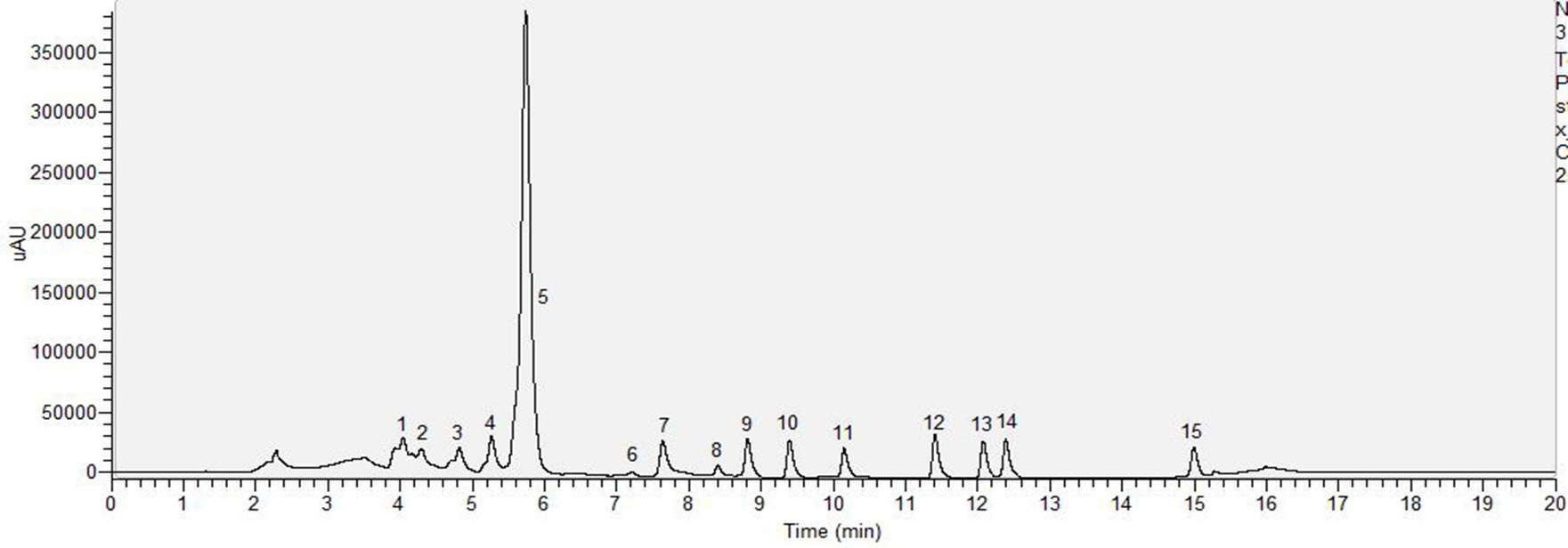


(A)


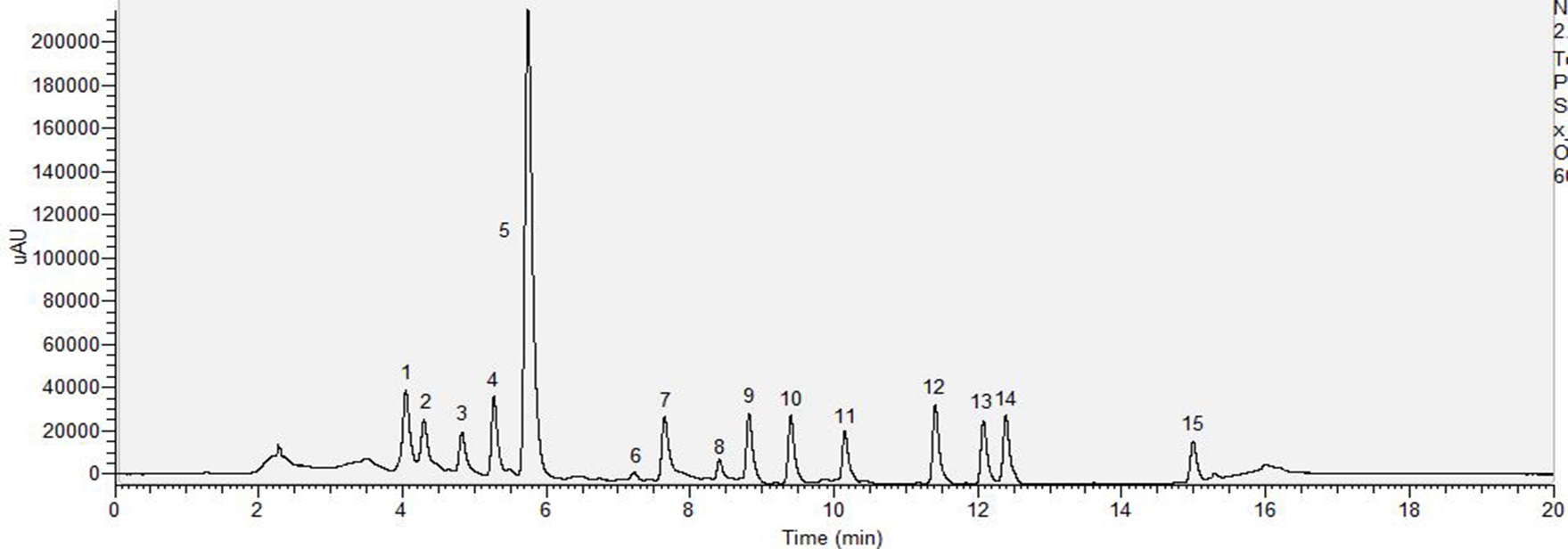


(B)

**Additional file 1: Figure S1** HPLC profile of carotenoid standards recorded in the range of 250.00-700.00 nm in different injection solvents. (**A**) Standard mix dissolved in MeOH:MTBE (25:75) and (**B**) Standard mix dissolved in MeOH:MTBE (60:40) The compounds are (1) violaxanthin; (2) neoxanthin; (3) anthraxanthin; (4) lutein; (5) zeaxanthin; (6) phytoene; (7) β-cryptoxanthin; (8) phytofluene; (9) α-carotene; (10) β-carotene; (11) ζ-carotene; (12) δ-carotene; (13) γ-carotene; (14) neurosporene; (15) lycopene.
