# Supplementary material for: A rapid and sensitive method for determination of carotenoids in plant tissues by high performance liquid chromatography
Source: Plant Methods. 2015 Feb 6;11:5. doi: 10.1186/s13007-015-0051-0 (PMC4329677; doi:10.1186/s13007-015-0051-0)
Supplement: Additional file 2: Figure S2. — On-line PDA spectra of all-trans and photoisomerised standards. (1) 9′-cis β-cryptoxanthin , (2) 9′-cis α-carotene, (3) 9′-cis antheraxanthin, (4) 9-cis β-cryptoxanthin, (5) 9-cis lutein, (6) 9-cis zeaxanthin, (7) 9-cis α-carotene, (8) 9-cis antheraxanthin, (9) 9-cis β-carotene, (10) 13-cis α-carotene, (11) 13′-cis α-carotene, (12) 13-cis β-cryptoxanthin, (13) 13′-cis β-cryptoxanthin, (14) 13-cis neurosporene, (15) 13 or 13′-cis lutein, (16) 13-cis zeaxanthin, (17) 15-cis neurosporene, (18) 15-cis β-carotene, (19) 15-cis zeaxanthin, (20) all-trans α-carotene, (21) all-trans antheraxanthin, (22) all-trans β-carotene, (23) all-trans β-cryptoxanthin, (24) all-trans δ-carotene, (25) all-trans γ-carotene, (26) all-trans lutein, (27) all-trans lycopene, (28) all-trans neoxanthin, (29) all-trans neurosporene, (30) all-trans violaxanthin, (31) all-trans zeaxanthin, (32) all-trans ζ-carotene, (33) cis- γ-carotene 2, (34) cis- γ-carotene 1, (35) cis-neoxanthin, (36) cis-violaxanthin, (37) phytoene isomer, (38) phytoene, (39) phytofluene isomer (40) phytofluene (41) di-cis lycopene, (42) di-cis lycopene, (43) 15-cis lycopene, (44) 13-cis lycopene, (45) 9-cis lycopene, (46) di-cis lycopene and (47) 5-cis lycopene. [file 13007_2015_51_MOESM2_ESM.docx]

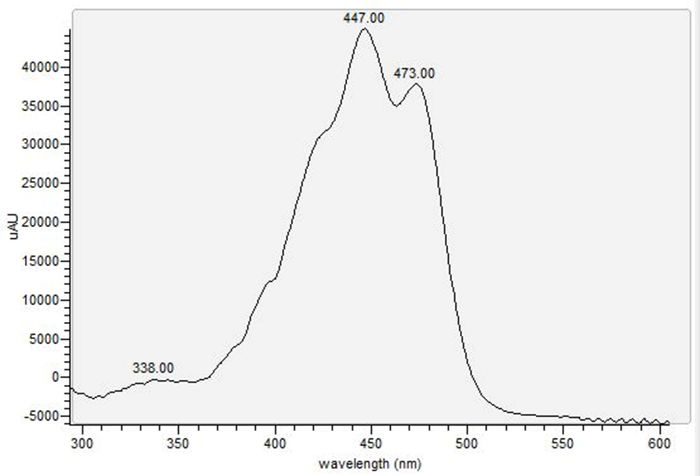

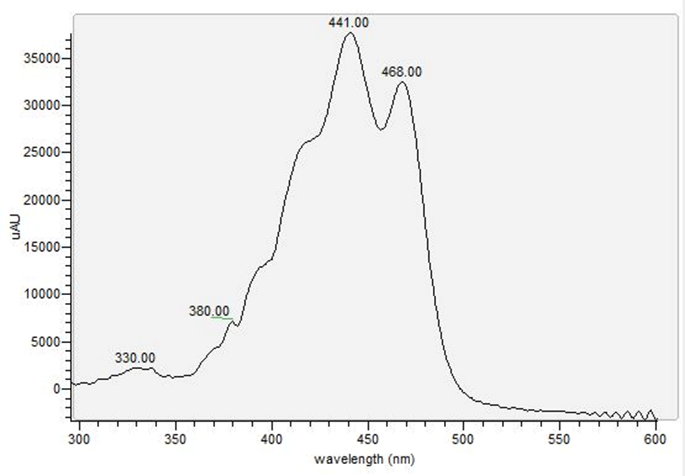

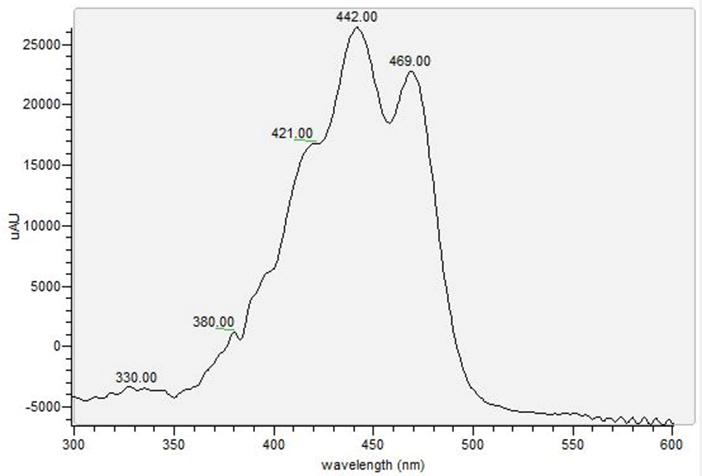

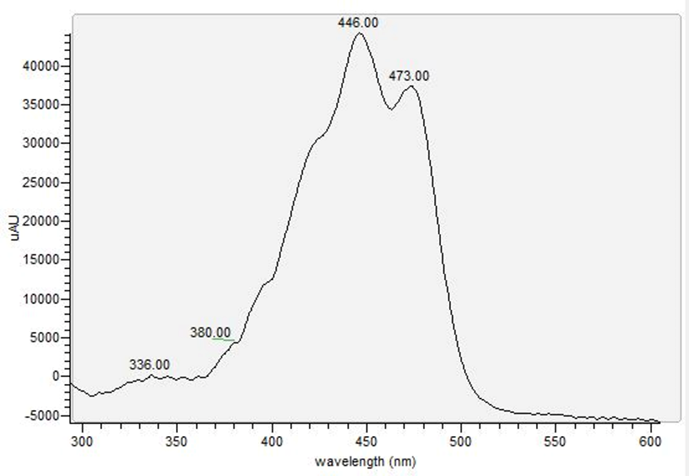

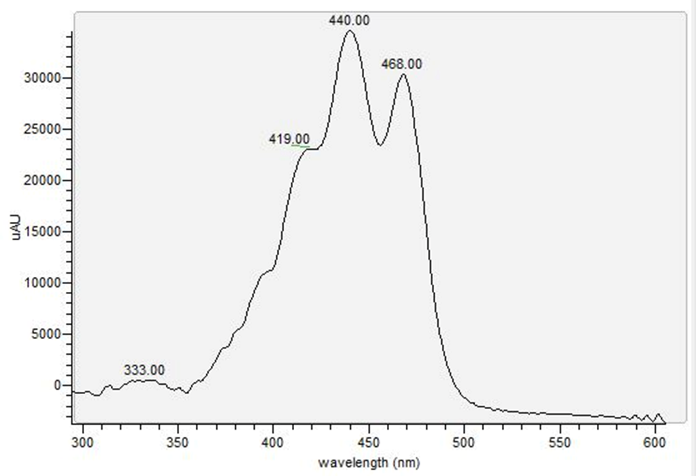

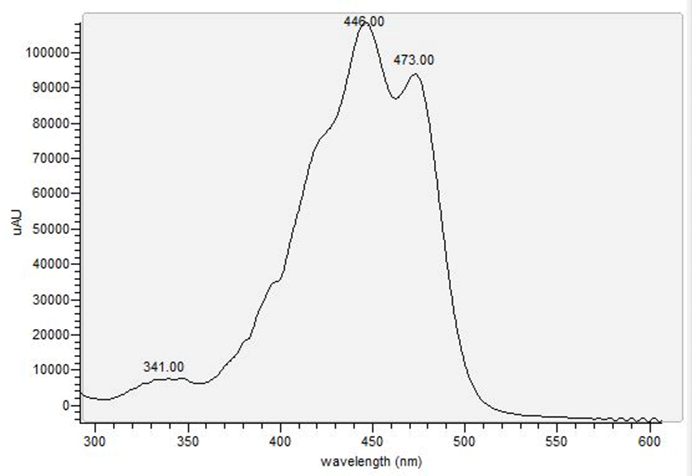

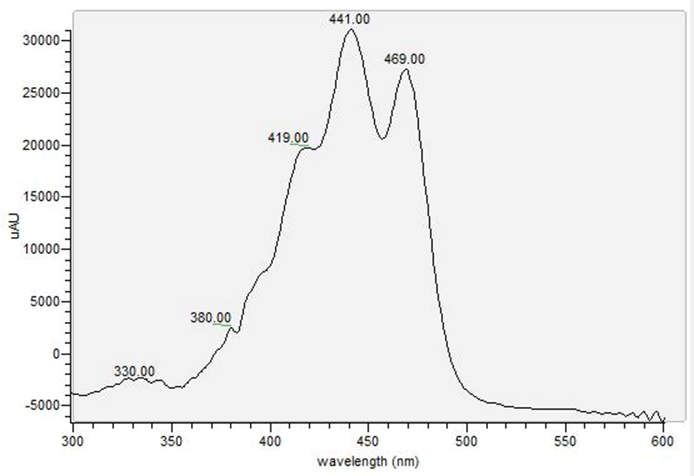

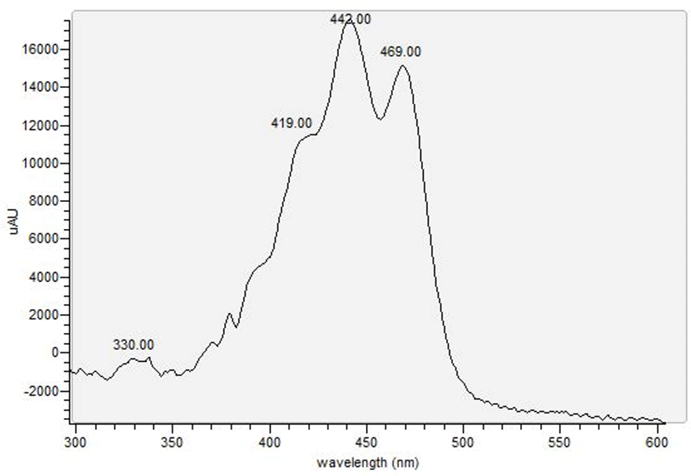


(7)

(8)

(5)

(6)

(3)

(4)

(2)

(1)


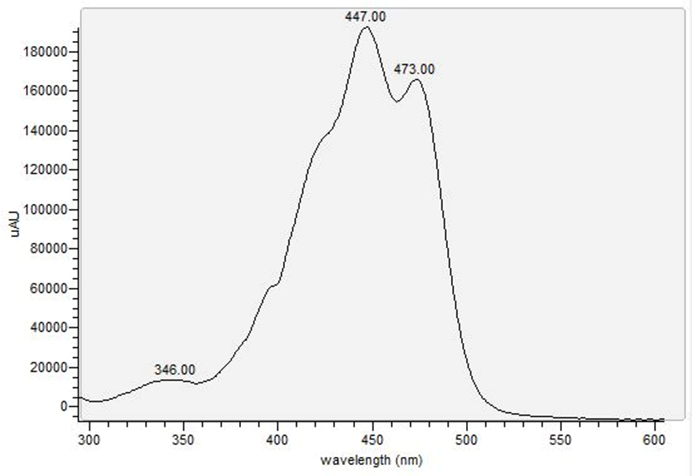

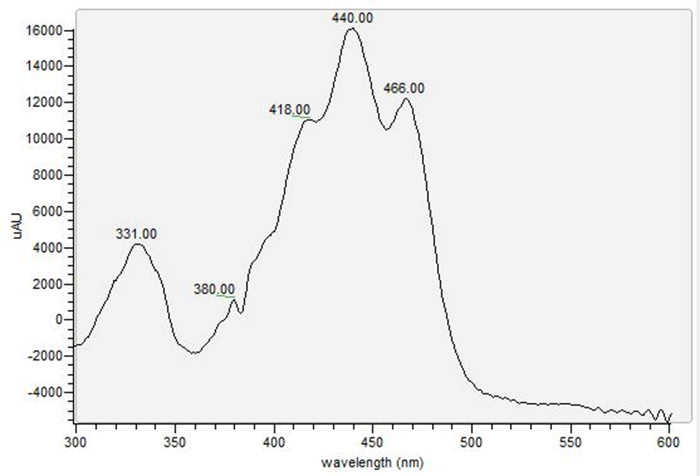

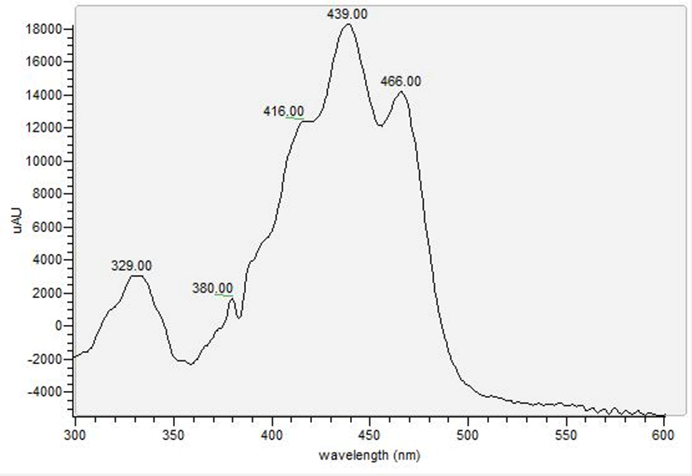

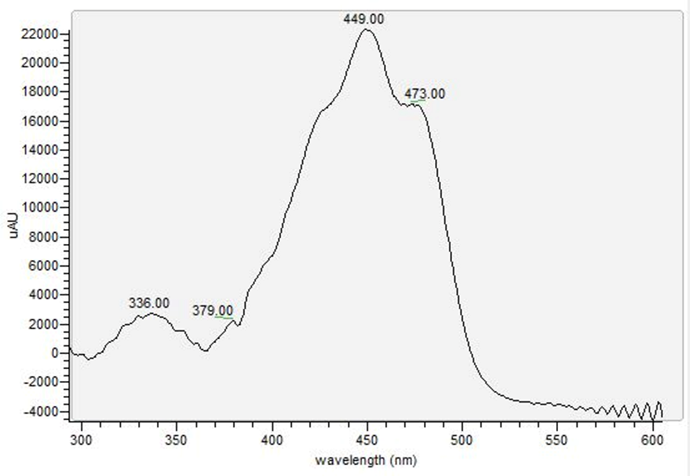

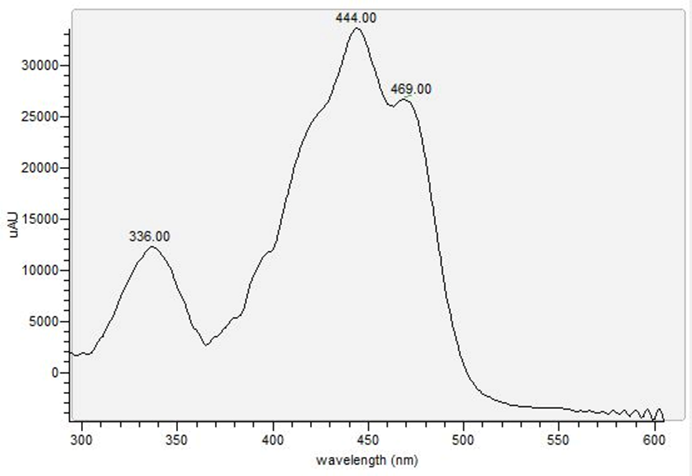

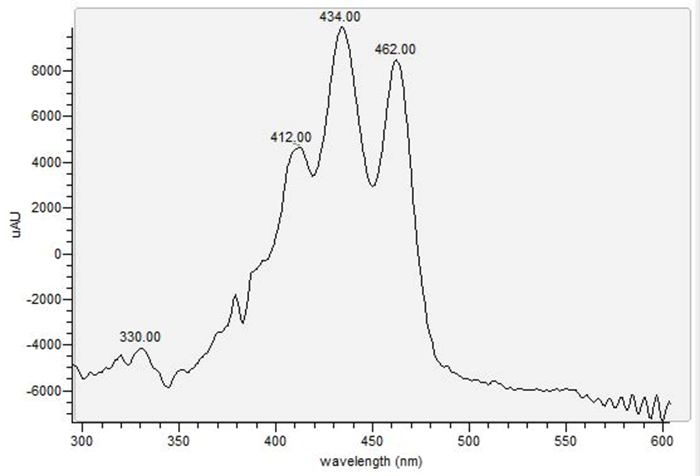

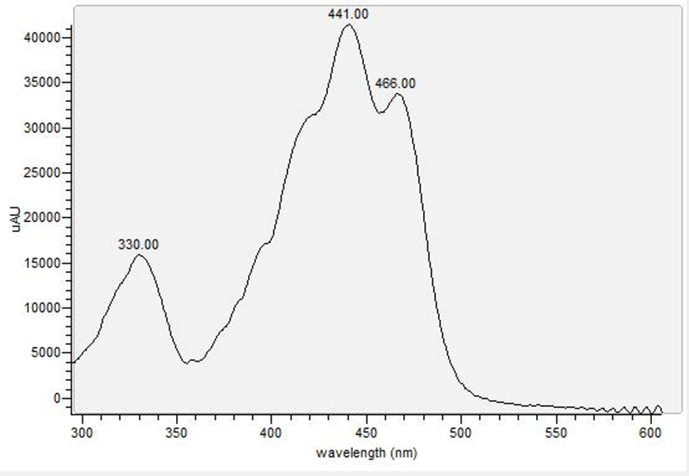

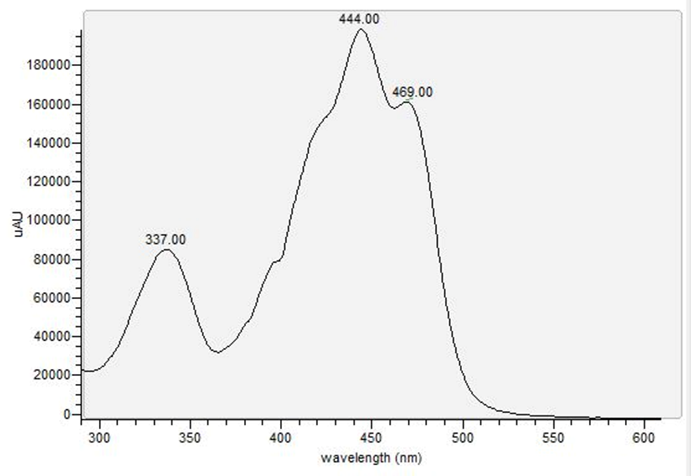


(16)

(15)

(14)

(13)

(12)

(11)

(10)

(9)


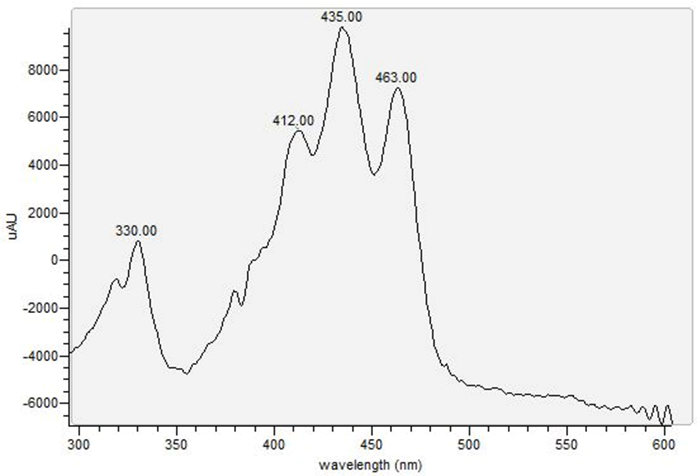

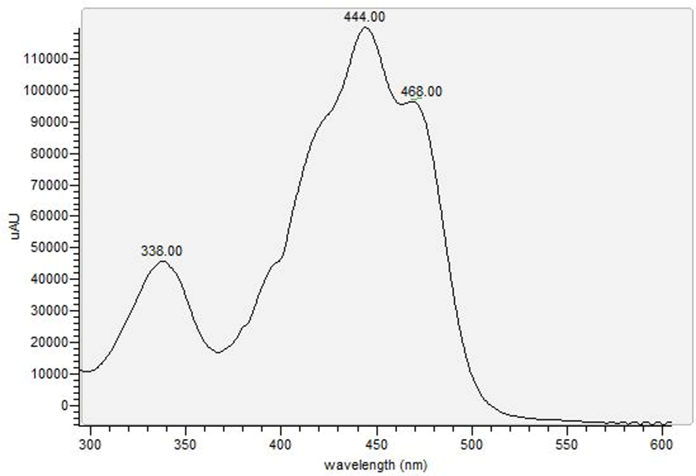

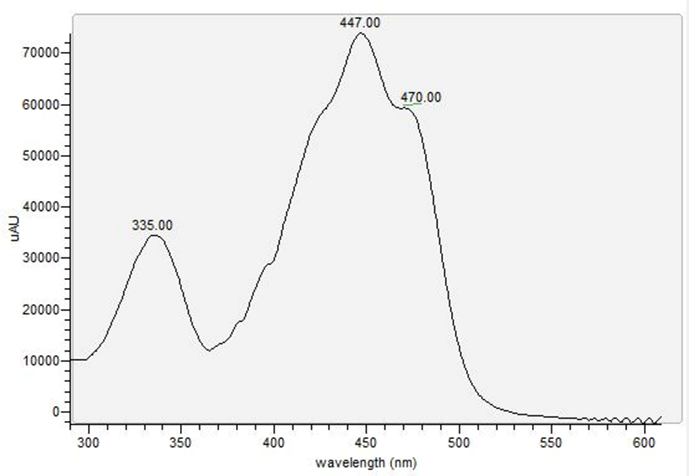

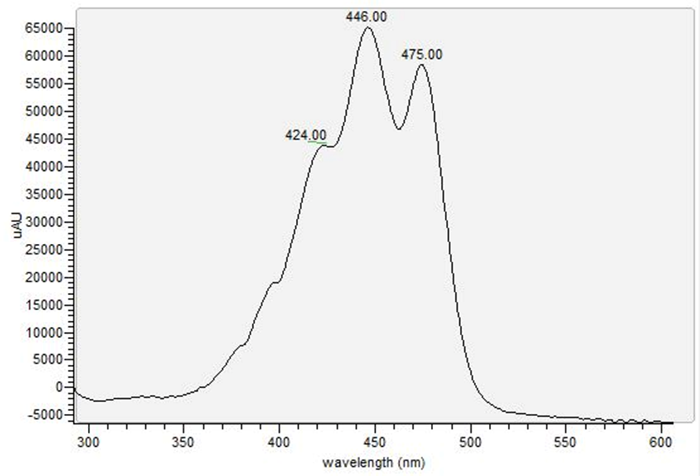

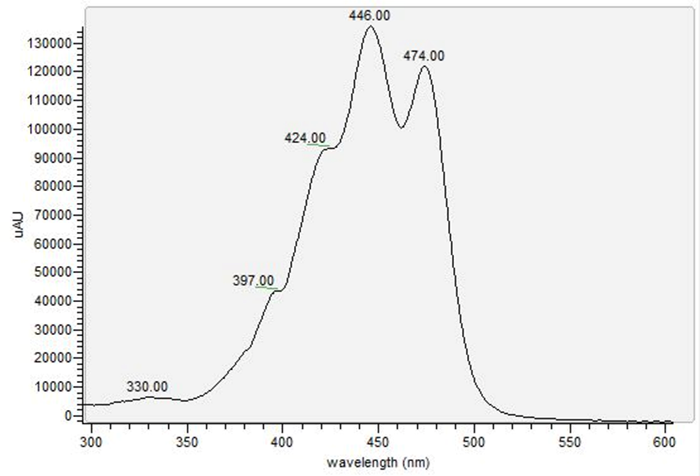

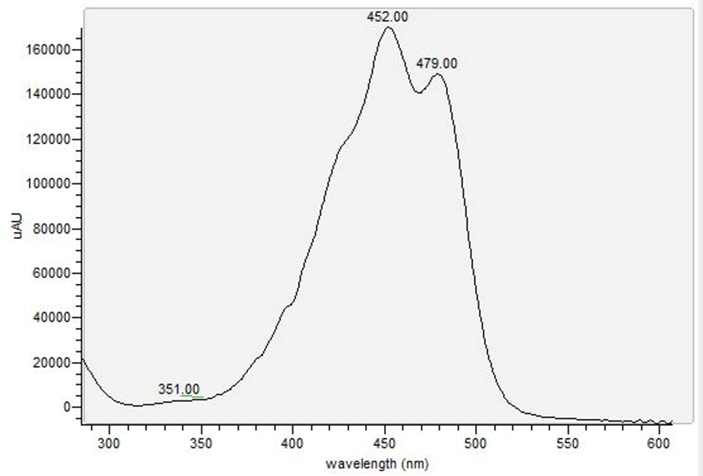

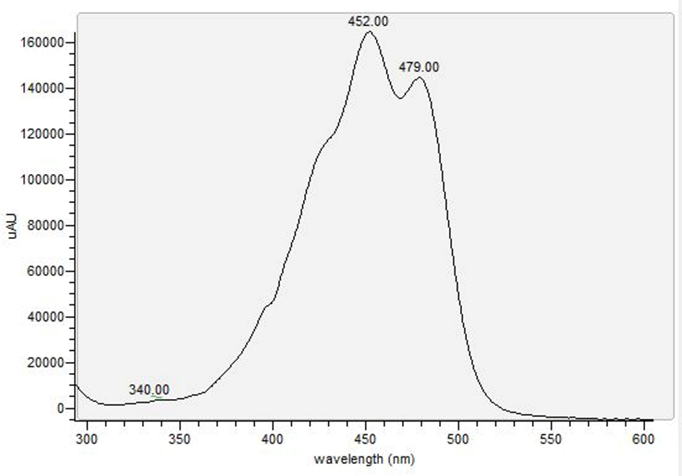

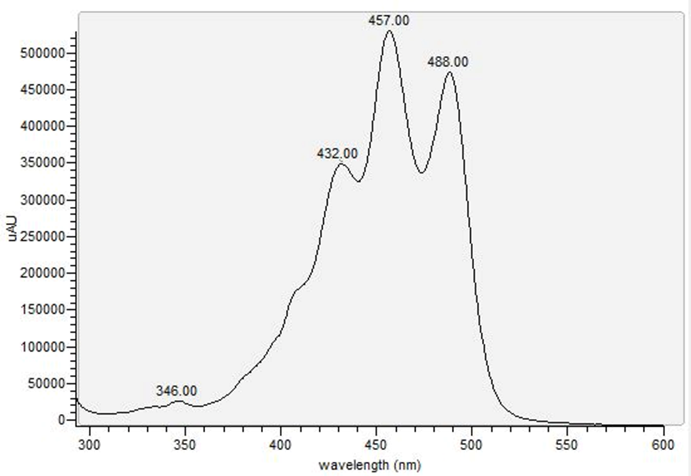


(24)

(23)

(22)

(21)

(19)

(20)

(18)

(17)


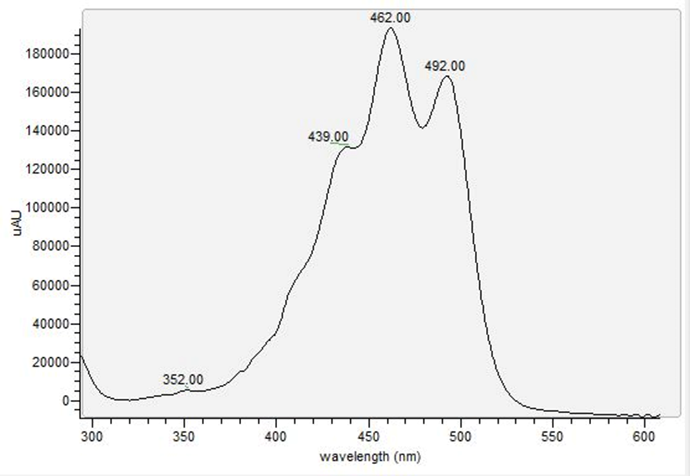

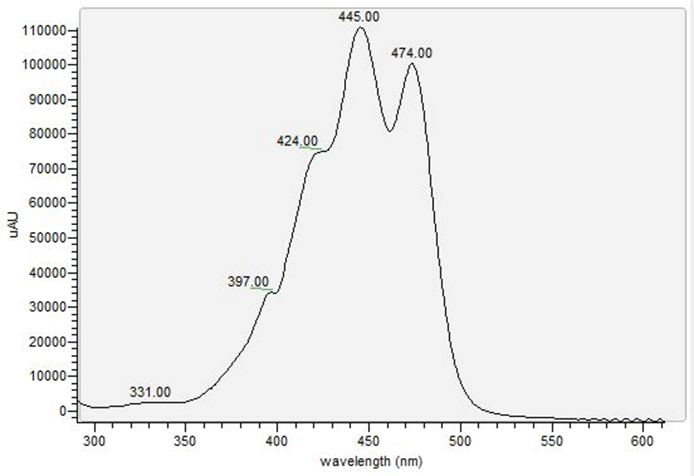

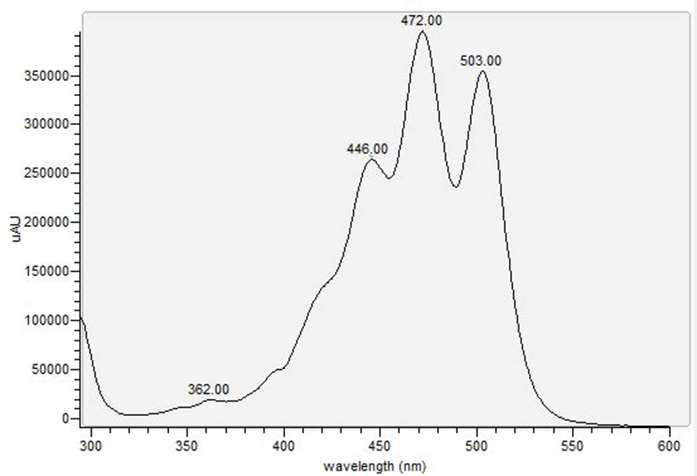

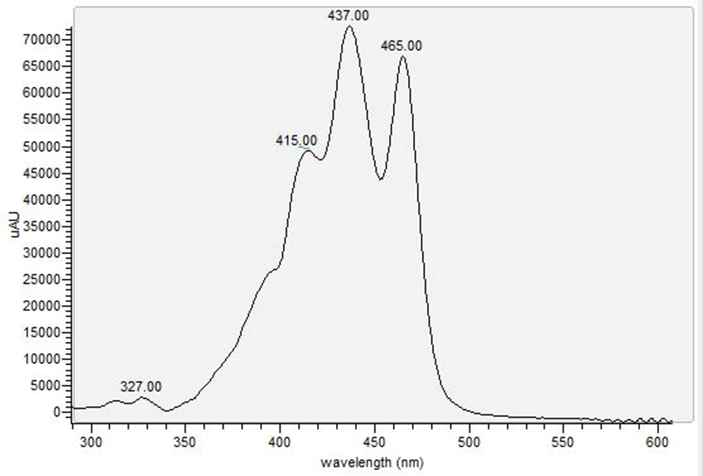

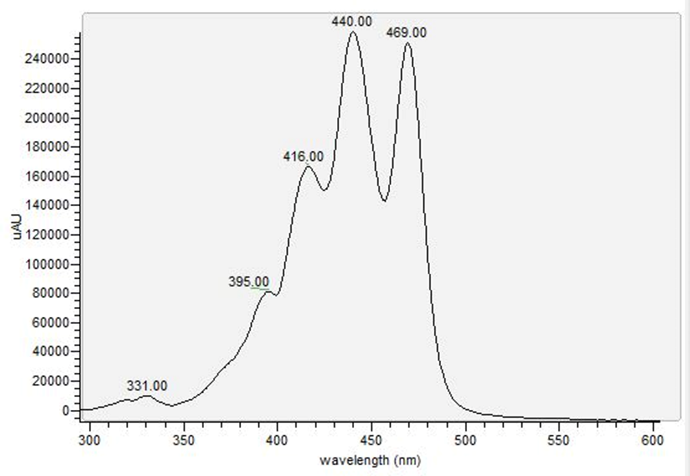

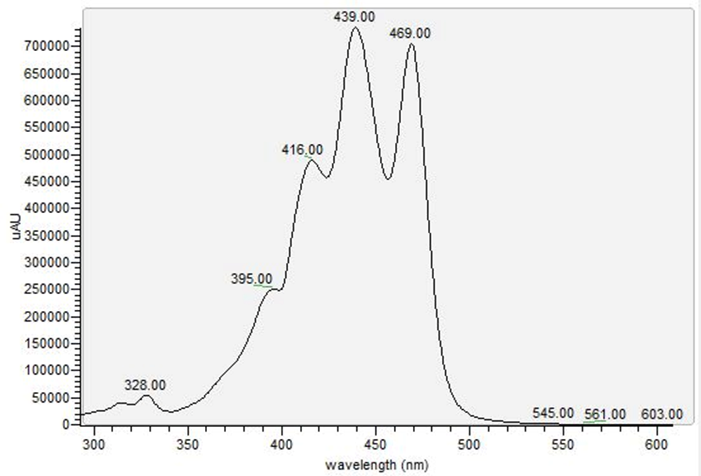

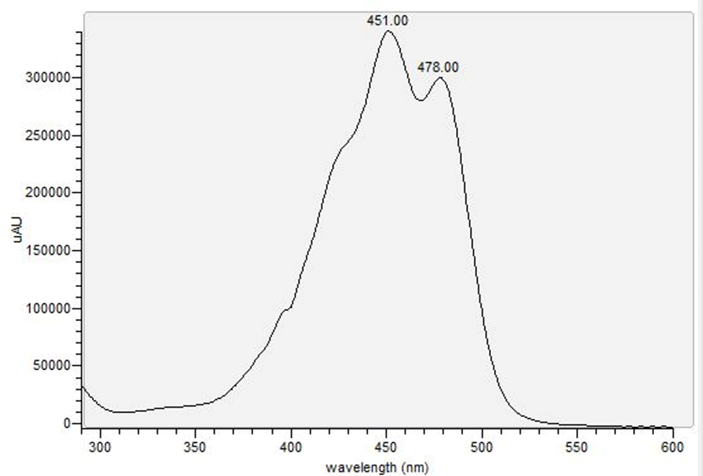

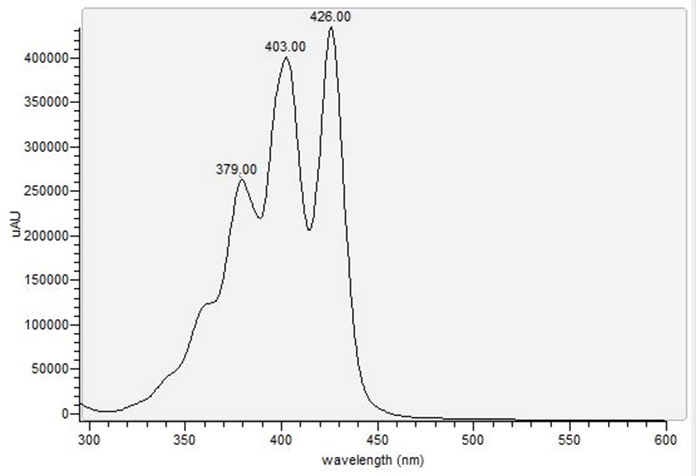


(32)

(31)

(30)

(29)

(28)

(27)

(26)

(25)


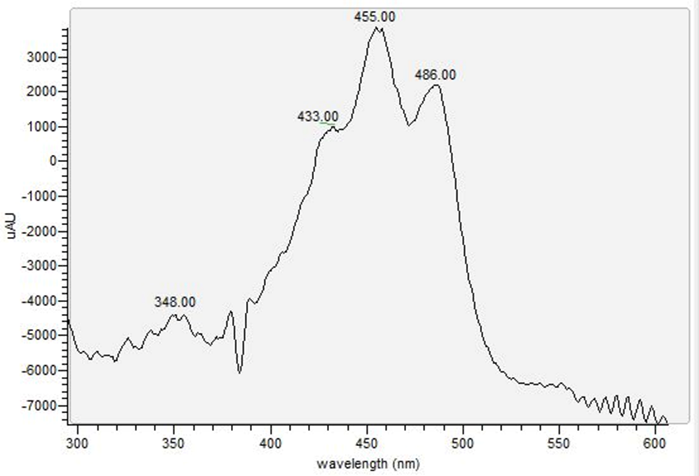

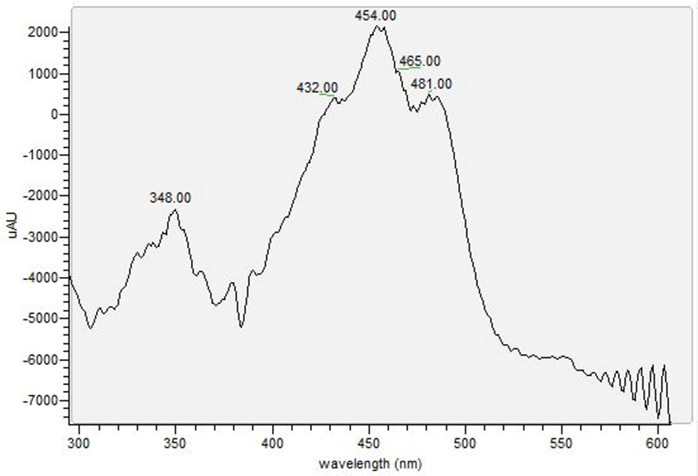

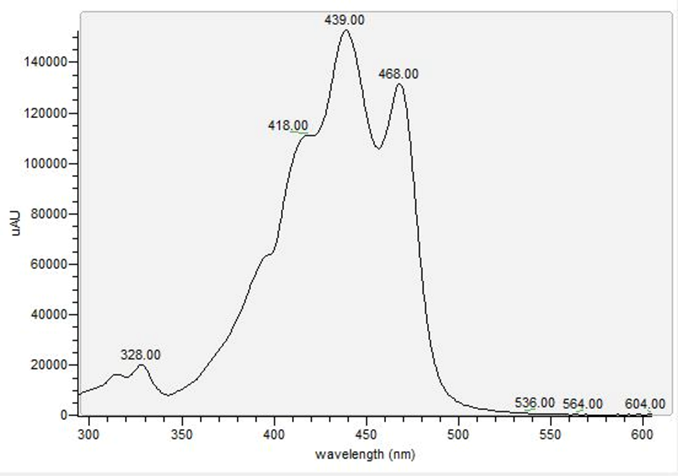

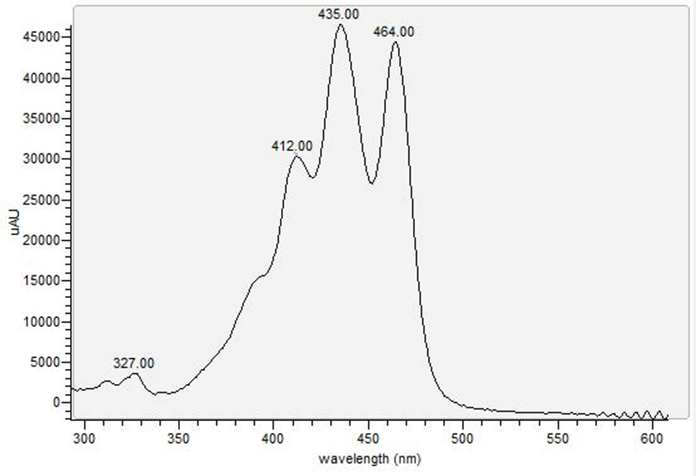

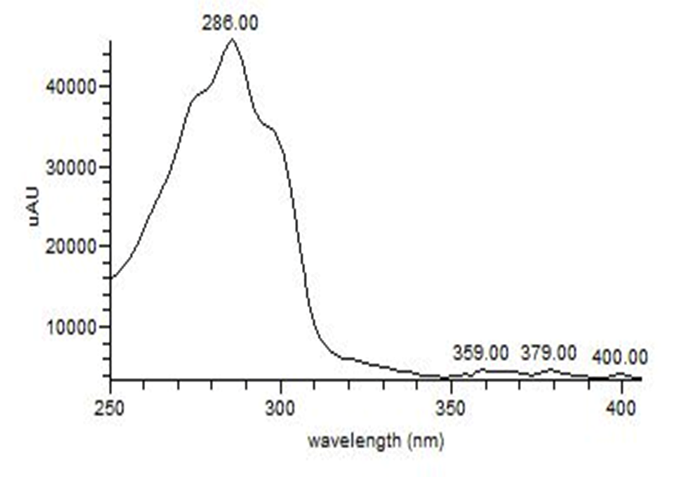

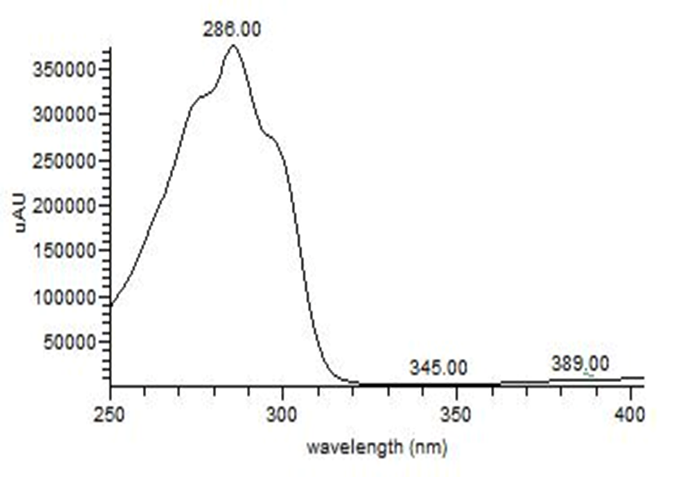

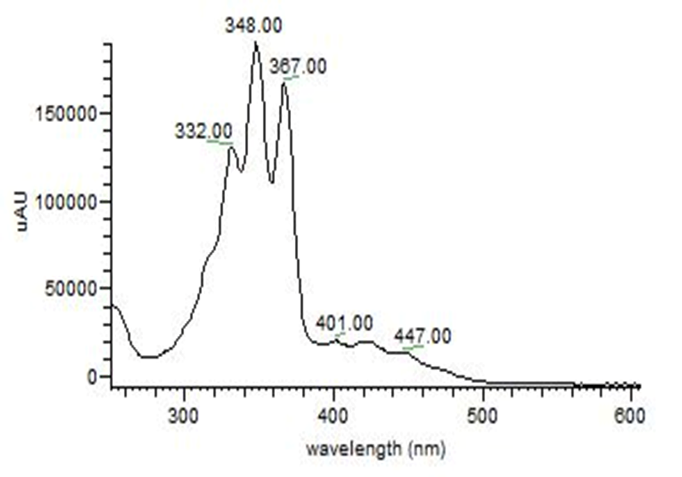

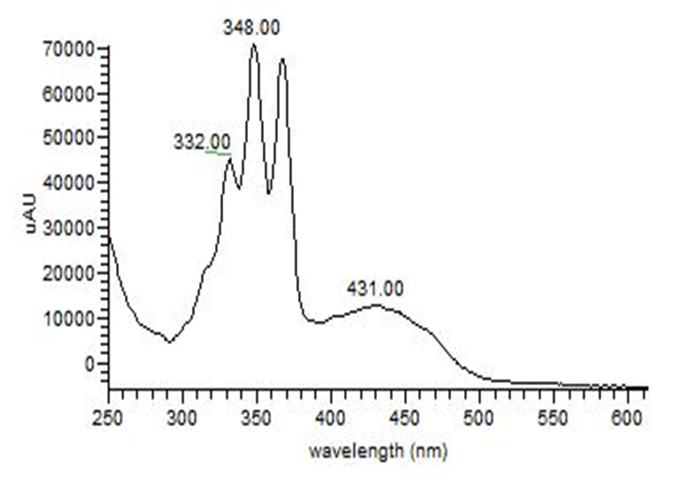


(40)

(40)

(39)

(38)

(37)

(36)

(35)

(34)

(33)


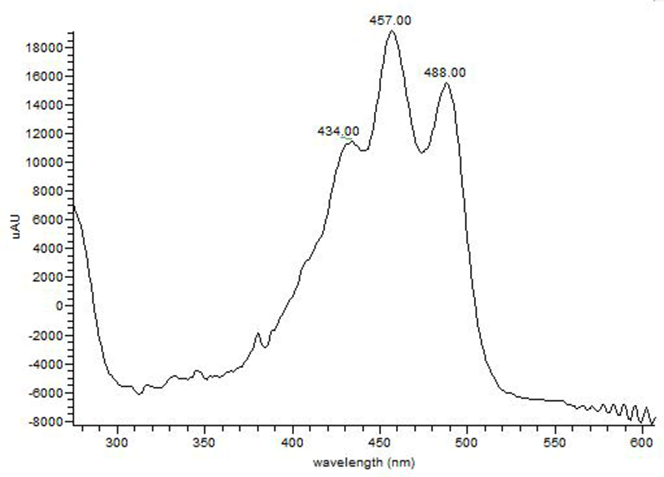

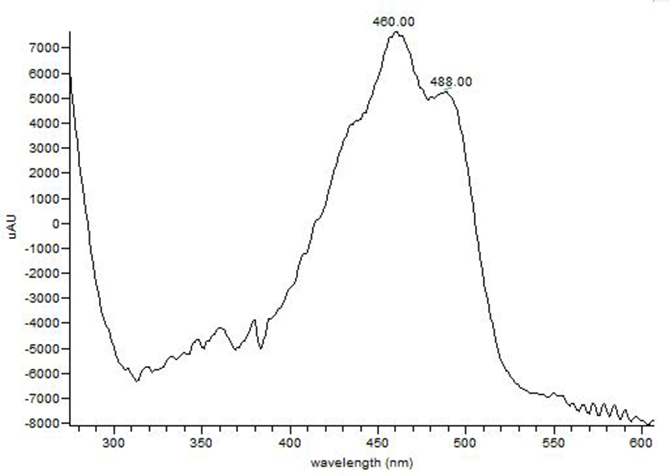

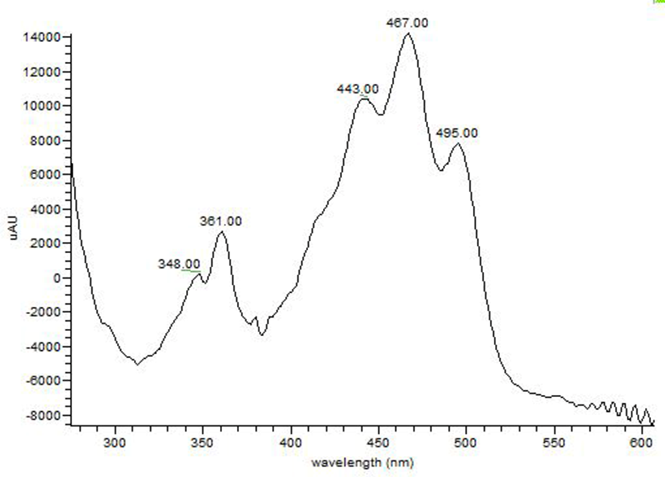

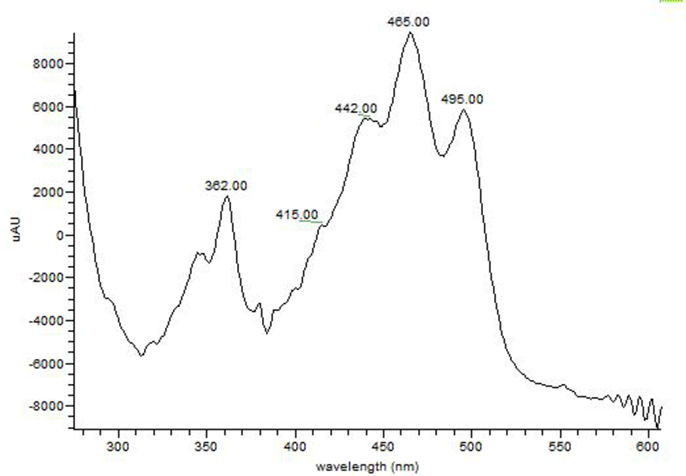

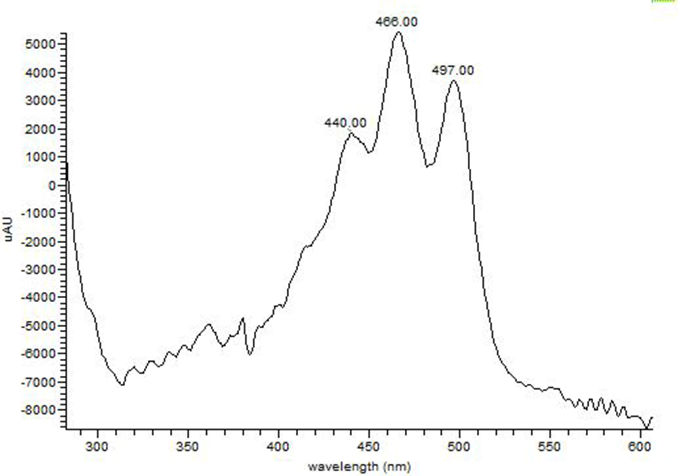

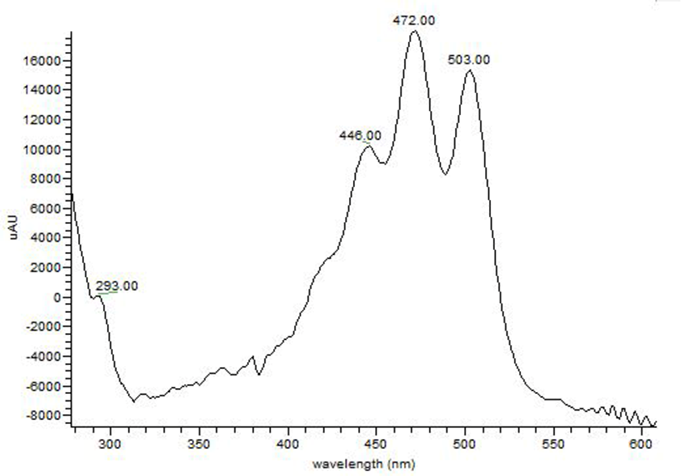


(46)

(45)

(43)

(42)

(44)

(41)


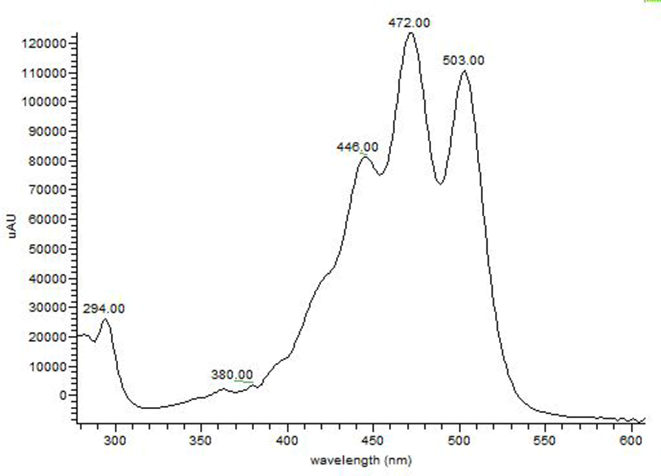


(47)

**Additional file 2: Figure S2.** On-line PDA spectra of all-trans and photoisomerised standards. (1) 9’-cis β-cryptoxanthin , (2) 9’-cis α-carotene, (3) 9’-cis antheraxanthin, (4) 9-cis β-cryptoxanthin, (5) 9-cis lutein, (6) 9-cis zeaxanthin, (7) 9-cis α-carotene, (8) 9-cis antheraxanthin, (9) 9-cis β-carotene, (10) 13-cis α-carotene, (11) 13’-cis α-carotene, (12) 13-cis β-cryptoxanthin, (13) 13’-cis β-cryptoxanthin, (14) 13-cis neurosporene, (15) 13 or 13’-cis lutein, (16) 13-cis zeaxanthin, (17) 15-cis neurosporene, (18) 15-cis β-carotene, (19) 15-cis zeaxanthin, (20) all-trans α-carotene, (21) all-trans antheraxanthin, (22) all-trans β-carotene, (23) all-trans β-cryptoxanthin, (24) all-trans δ-carotene, (25) all-trans γ-carotene, (26) all-trans lutein, (27) all-trans lycopene, (28) all-trans neoxanthin, (29) all-trans neurosporene, (30) all-trans violaxanthin, (31) all-trans zeaxanthin, (32) all-trans ζ-carotene, (33) cis- γ-carotene 2, (34) cis- γ-carotene 1, (35) cis-neoxanthin, (36) cis-violaxanthin, (37) phytoene isomer, (38) phytoene, (39) phytofluene isomer (40) phytofluene (41) di-cis lycopene, (42) di-cis lycopene, (43) 15-cis lycopene, (44) 13-cis lycopene, (45) 9-cis lycopene, (46) di-cis lycopene and (47) 5-cis lycopene.
