# Supplementary material for: A rapid and sensitive method for determination of carotenoids in plant tissues by high performance liquid chromatography
Source: Plant Methods. 2015 Feb 6;11:5. doi: 10.1186/s13007-015-0051-0 (PMC4329677; doi:10.1186/s13007-015-0051-0)
Supplement: Additional file 3: Table S1. — Absorption Coefficients of carotenoid standards. [file 13007_2015_51_MOESM3_ESM.doc]

**Additional file 3: Table S1**. Absorption Coefficients of carotenoid standards

| **Compound** | **Solvent** | **λ (nm)** | **A (1%, 1cm)** |
| --- | --- | --- | --- |
| All-trans-violaxanthin | Ethanol | 440 | 2561 |
| All-trans-neoxanthin | Ethanol | 437 | 2253 |
| All-trans-antheraxanthin | Ethanol | 447 | 2285 |
| All-trans lutein | Ethanol | 445 | 2550 |
| All-trans-zeaxanthin | Ethanol | 450 | 2540 |
| 15-cis-phytoene | Hexane/2% CH_2_Cl_2_ | 286 | 760 |
| All-trans- β-cryptoxanthin | Hexane/2% CH_2_Cl_2_ | 450 | 2593 |
| All-trans-phytofluene | Hexane/2% CH_2_Cl_2_ | 348 | 1657 |
| All-trans- α-carotene | Hexane/2% CH_2_Cl_2_ | 444 | 2895 |
| All-trans- β-carotene | Hexane/2% CH_2_Cl_2_ | 450 | 2572 |
| All-trans- ζ-carotene | Hexane/2% CH_2_Cl_2_ | 425 | 2617 |
| All-trans- δ-carotene | Hexane/2% CH_2_Cl_2_ | 455 | 3221 |
| All-trans- γ-carotene | Hexane/2% CH_2_Cl_2_ | 460 | 3149 |
| All-trans-neurosporene | Hexane/2% CH_2_Cl_2_ | 439 | 2938 |
| All-trans-lycopene | Hexane/2% CH_2_Cl_2_ | 471 | 3530 |
